# A Chromosome-level Genome Assembly of Wild Castor Provides New Insights into its Adaptive Evolution in Tropical Desert

**DOI:** 10.1016/j.gpb.2021.04.003

**Published:** 2021-07-30

**Authors:** Jianjun Lu, Cheng Pan, Wei Fan, Wanfei Liu, Huayan Zhao, Donghai Li, Sen Wang, Lianlian Hu, Bing He, Kun Qian, Rui Qin, Jue Ruan, Qiang Lin, Shiyou Lü, Peng Cui

**Affiliations:** 1 CAS Key Laboratory of Plant Germplasm Enhancement and Specialty Agriculture, Wuhan Botanical Garden, Chinese Academy of Sciences, Wuhan 430074, China; 2 Shenzhen Branch, Guangdong Laboratory for Lingnan Modern Agriculture, Genome Analysis Laboratory of the Ministry of Agriculture and Rural Affairs, Agricultural Genomics Institute at Shenzhen, Chinese Academy of Agricultural Sciences, Shenzhen 518120, China; 3 University of Chinese Academy of Sciences, Beijing 100049, China; 4 Sino-Africa Joint Research Center, Chinese Academy of Sciences, Wuhan 430074, China; 5 State Key Laboratory of Biocatalysis and Enzyme Engineering, School of Life Sciences, Hubei University, Wuhan 434200, China

**Keywords:** *Ricinus communis* L., Adaptive evolution, Selection signature, Genetic variation, Genome assembly

## Abstract

Wild castor grows in the high-altitude tropical desert of the African Plateau, a region known for high ultraviolet radiation, strong light, and extremely dry condition. To investigate the potential genetic basis of adaptation to both highland and tropical deserts, we generated a chromosome-level genome sequence assembly of the wild castor accession WT05, with a genome size of 316 Mb, a scaffold N50 of 31.93 Mb, and a contig N50 of 8.96 Mb, respectively. Compared with cultivated castor and other Euphorbiaceae species, the wild castor exhibits positive selection and gene family expansion for genes involved in DNA repair, photosynthesis, and abiotic stress responses. **Genetic variations** associated with positive selection were identified in several key genes, such as *LIG1*, *DDB2*, and *RECG1*, involved in nucleotide excision repair. Moreover, a study of genomic diversity among wild and cultivated accessions revealed genomic regions containing **selection signatures** associated with the adaptation to extreme environments. The identification of the genes and alleles with selection signatures provides insights into the genetic mechanisms underlying the adaptation of wild castor to the high-altitude tropical desert and would facilitate direct improvement of modern castor varieties.

## Introduction

Castor (*Ricinus communis* L.) is one of the most important oil crops worldwide. Castor seeds contain up to 65% oil content, of which approximately 90% has been identified as a hydroxy fatty acid named ricinoleic acid. Due to the multiple industry applications of ricinoleic acid, castor as an ideal bioenergy plant warranting the title of “green petroleum”, was first domesticated from a wild ancestor in Africa approximately 1000 years ago and then spread to Asia and America [1]. Wild castor still grows in the tropical desert area of the African Plateau at an altitude of more than 2000 m [2], [3]. This region exhibits extreme dryness, intense light, and ultraviolet (UV) radiation all year round. It acts as a natural laboratory for the study of species adaptation evolution. Wild castor plants have evolved a strong ability to adapt to extremely harsh conditions during genomic evolution. These treasured characteristics provide an ideal background for studying the adaptive evolution of the castor genome and the advantageous genetic resources for castor improvement.

Wild species resources play an indispensable role in the study of adaptive evolution, resistance mechanisms, and variety improvement. Till now, numerous studies have shown that wild species resources of different crops provide abundant germplasm resources and information regarding genetic variation for species research. Selection pressure analysis of wild and cultivated varieties has enabled to identify candidate genes that are associated with economic traits, such as the salt tolerance gene *GmCHX1* [4] and the seed coat-determining locus [5] in soybean. Photosynthetic efficiency-related genes undergoing positive selection have been identified in wild pear [6]. Pathogen- and abiotic stress-related genes have been identified in wild cassava [7]. African wild rice species have donated some candidate genes for resistance to biotic stresses [8]. All of the aforementioned genes respond to the wild ecological niche and have undergone strong selections after domestication procedures. These selection signatures provide an important reference for functional genomics and novel insights into adaptive evolution and crop improvement.

**Figure 1:**
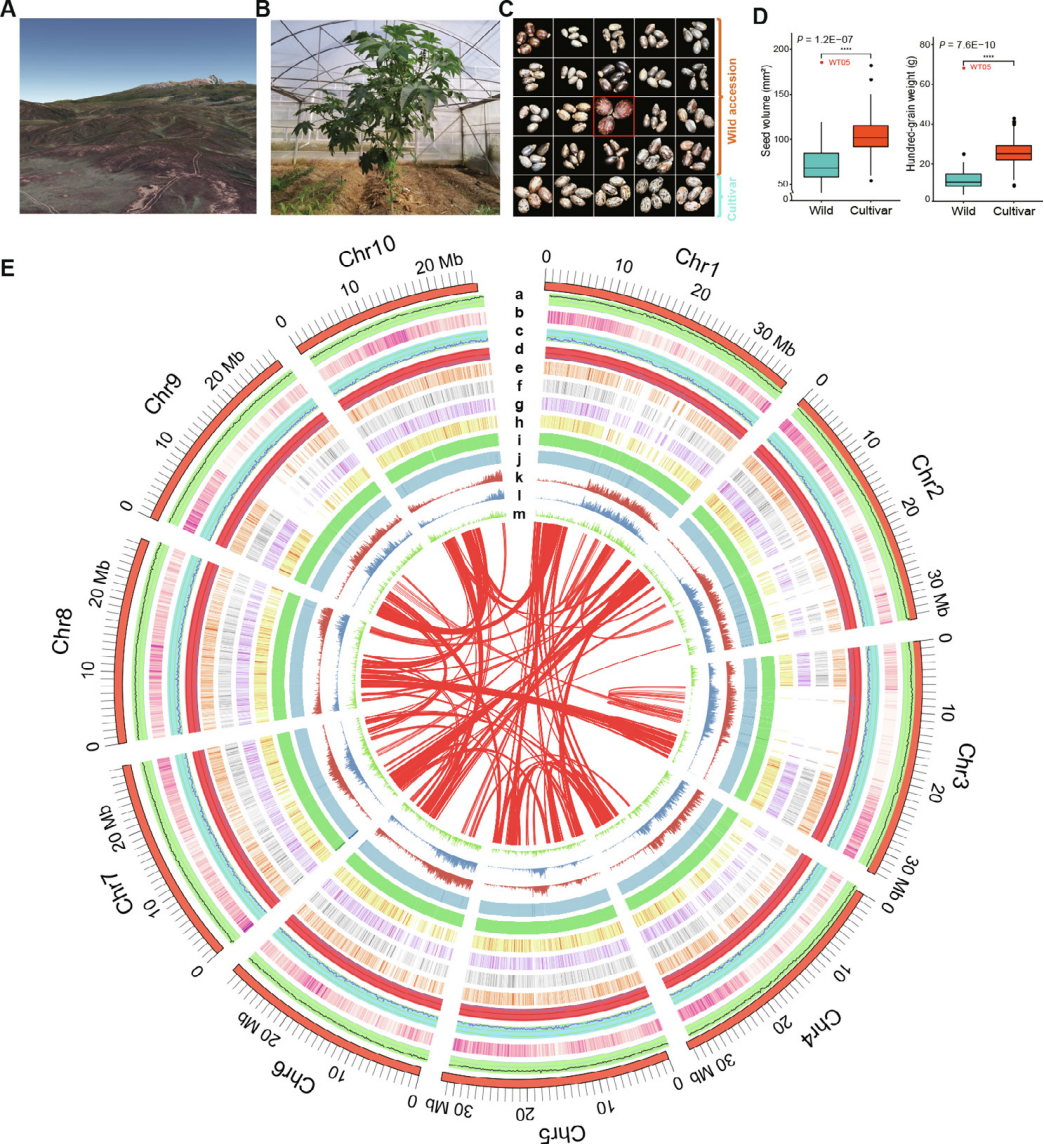
**Distribution of genomic features along the castor genome** **A.** A glimpse of Kenya National Park (Google earth v2020). **B.** Picture of the representative wild castor growing in the arid regions of Africa. **C.** Comparison of seed diversity between wild and cultivated varieties. **D.** Statistical analysis of castor seed differences between wild and cultivated castor varieties in China. The centerline marks the median. Box limits are upper and lower quartiles. Whiskers extend to data less than 1.5 times the interquartile range. Dots represent outliers. Red font (WT05) represents the wild-type variety that was selected for assembly. Wilcoxon test (****, *P* < 0.00005). **E.** Characteristics of the WT05 genome. The rings from outer to inner represent (a) GC density, (b) gene density, (c) indel, (d) SNP diversity, (e–h) gene expression levels in root, stem, leaf, and seed, respectively (Log_10_ TPM), (i) Nanopore reads mapping depth, (j) NGS reads mapping depth, (k) LTR-RT distribution, (l) *Gypsy* distribution, and (m) *Copia* distribution. Central colored lines represent syntenic links. In a–d, i–k, and m, statistics are based on window size of 100 kb. Indel, insertion and deletion; SNP, single nucleotide polymorphism; TPM, transcripts per kilobase of exon model per million mapped reads; NGS, next generation sequencing; LTR-RT, long-terminal repeat retrotransposon.

In this study, we first collected and identified a superior wild castor (WT05) from the center of castor origin in Africa (Figure 1A–D). To investigate genetic mechanisms that are associated with environmental adaptability in castor WT05, we integrated multifaceted sequencing and assembly approach using a combination of Oxford Nanopore technology and three-dimensional chromosome conformation capture (Hi-C) sequencing to obtain a chromosome-scale genome of castor WT05, which greatly improved the quality of the reference genome and provided precise genomic information for studies on castor. Through comparative genomic and evolutionary analyses with an inbred cultivar genome NSL4733 (Hale) published in 2010 [9] and four other Euphorbiaceae plant genomes (Table S1) that have been sequenced to date, we showed that a great number of genes, involving in pathways of DNA repair, photosynthesis, and stress responses, have undergone positive natural selection, which is closely associated with adaptation to highland and tropical desert environments. Our work reveals the genetic basis of the adaptation of wild castor to tropical deserts and provides a set of genes and alleles for future molecular breeding and improvement.

## Results

### *De novo* assembly and annotation of the wild castor genome

We totally generated 3.86 million long reads with a total length of 61.58 Gb (average read length 15.95 kb), representing ~ 170× sequencing coverage of the reference genome (Table S2). Initial assembly of 315.95 Mb contains 301 contigs with the contig N50 length of 8.96 Mb and the largest contig of 27.25 Mb. The genome size is close to the 25-mer estimation of ~ 318.13 Mb (Figure S1) and slightly less than the cultivar reference genome (350 Mb for cultivar NSL4733 published in 2010) [9]. Approximately 74.6 Gb Hi-C data were generated to achieve the final chromosomal-level assembling (Figure S2). The final size of the assembly is 316.11 Mb, of which 311.90 Mb (98.67%) was anchored onto 10 chromosome-level scaffoldings (Figure 1E). The sizes of the 10 chromosomes vary from 26.62 Mb to 36.69 Mb. Long-terminal repeat (LTR) Assembly Index (LAI) of the genome was calculated to be 10.54, suggesting that it could be served as a reference genome. Statistics of this genome assembly showed much more superiority than the cultivar reference in continuity and integrality (Table 1).

**Table 1:** **Statistics of the genome assemblies**

**Assembly**	**WT05_contig**	**WT05_scaffold**	**NSL4733_scaffold**
Number of sequences	301	146	25,828
Number of sequences (≥ 50,000 bp)	293	37	891
Number of genes	–	30,066	31,221
Number of mRNAs	–	43,272	31,221
Total length (bp)	315,948,298	316,113,298	350,631,014
Largest contig (bp)	27,252,567	36,693,184	4,693,355
N50 length (bp)	8,963,070	31,927,722	496,528
GC content (%)	33.05	33.05	33.84
Repeat sequence content (%)	–	52.95	53.35
L50	12	5	167
Number of N's per 100 kb	–	52.20	3896.61

*Note*: “–” indicates unannotated.

To evaluate the completeness of the newly assembled draft genome, a total of 133,384,288 Illumina paired-end reads, with a size of nearly 20.0 Gb (Table S3), and 3,860,238 Nanopore raw reads were aligned to the newly assembled genome, 96.76% and 84.49% of the reads were successfully aligned to the genome, respectively. Then, the completeness of genes was further assessed using 1440 Benchmarking Universal Single-Copy Orthologs (BUSCO) [10] genes from Embryophyta, of which 1377 genes (95.63%) are complete conserved genes, including 1352 single-copy and 25 duplicated orthologous genes (Table S4). In addition, using transcriptome data from three WT05 tissues (including stem, leaf, and seed), 93.40%, 91.23%, and 98.51% of the reads could be aligned onto the draft genome sequence, respectively (Table S5). These results suggest that the newly assembled genome is of high quality.

In total, 30,066 protein-coding genes were predicted, and their functions were further annotated based on the Trembl, Non-Redundant Protein (NR), Swiss-Prot, InterPro, and KEGG databases (Table S6). 97.84% (29,418/30,066) of the genes were anchored in the 10 chromosomes. In addition, we identified and annotated different types of non-coding RNA sequences, including 579 miRNAs, 830 tRNAs, 159 rRNAs, and 1770 snRNAs (Table S7).

Transposable elements (TEs) play indispensable roles in genome evolution. We identified 167.37 Mb of repeat sequences that occupy 52.95% of the total genome length, slightly less than that reported for the previous reference genome NSL4733 (187.07 Mb, 53.35%). Long-terminal repeat retrotransposons (LTR-RTs) are the main components of TEs. In the genome of WT05, LTR-RTs mainly include *Gypsy* (21.10%) and *Copia* (4.90%) (Figure S3). Euphorbiaceae species show diversity in genome size distribution, varying from 316 Mb to 1.37 Gb (Table S1). Considering the extreme variations in genome size in Euphorbiaceae species, we investigated the dynamic changes in LTR-RTs during the evolution processes and tried to explain the large variations in the genome size of species in the Euphorbiaceae family. Wild or cultivated castor, compared with the other four important economic species of Euphorbiaceae, has a relatively small genome. LTR-RT proliferation occurred ~ 1.0 million years ago (MYA), and the most recent amplification was estimated to have occurred 0.2–0.5 MYA, according to the corresponding values of the highest sharp peak and foremost relatively minor fluctuating peak (Figure S4). More specifically, physic nut (*Jatropha curcas* L.; genome size = 416 Mb, 59.35%) [11] experienced another two short LTR-RT proliferations at 2.4 MYA and 3.6 MYA; cassava (*Manihot esculenta* Crantz; genome size = 582 Mb, 50.34%) [12] has a broader peak at ~ 1.0 MYA than castor (Figure S4). Additionally, for the tung tree (*Vernicia fordii*), with a G-scale genome size of 1.2 Gb and repeat sequence of 58.74%, we found that LTR-RTs remained active from 1.0 MYA to 2.0 MYA (Figure S4). Especially, the ratios of *Gypsy*-type LTR-RTs of the G-scale genomes of tung tree [13] and rubber tree (*Hevea brasiliensis*) are nearly twice that of castor (Table S1). Similarly, the genome study of desert poplar (*Populus trichocarpa*) also found that the widespread expansion of the *Gypsy* element has led to a rapid increase in the size of its genome [14]. Therefore, we infer that LTR-RT amplification leads to genome-size variations in Euphorbiaceae species.

### Comparative analysis of WT05 and NSL4733 genomes

**Figure 2:**
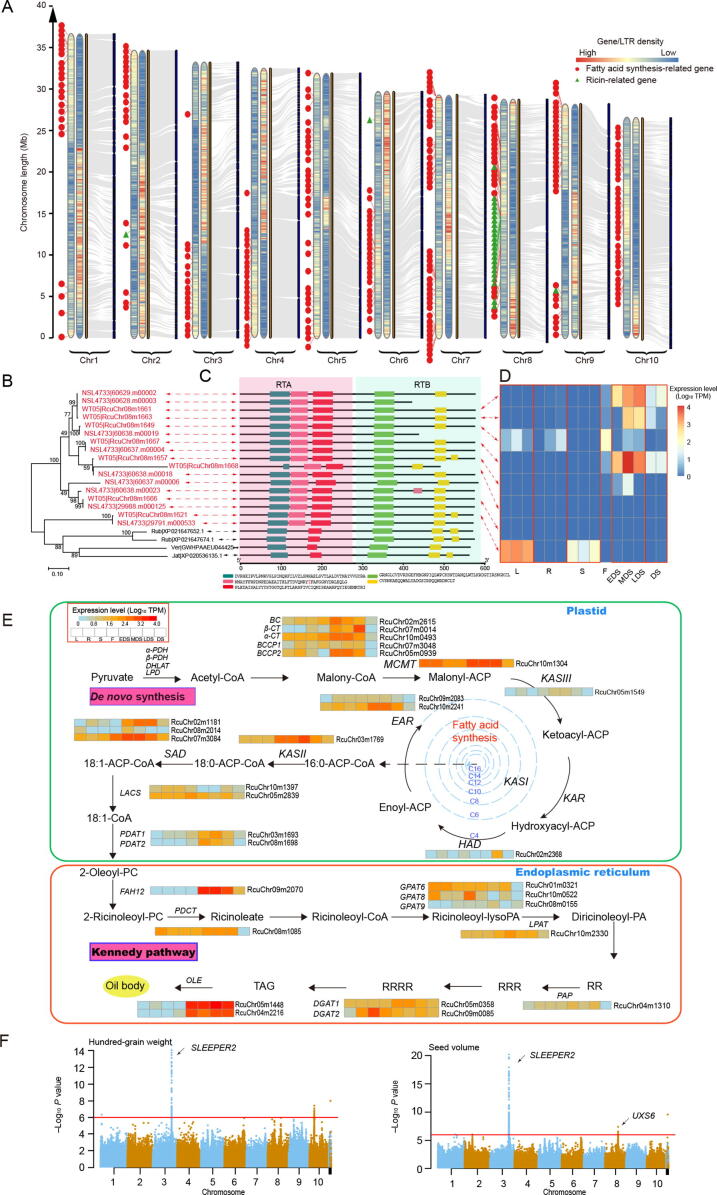
**Identification of ricin-relatedand fatty acid synthesis-related genes in WT05 genome** **A.** Genomic collinearity between the WT05 and NSL4733 genomes and the location of ricin-related and putative fatty acid synthesis-related genes in the whole genome. The two heat maps on each chromosome show the density distribution of genes (left) and repeats (right), respectively. Statistics are based on 100 kb non-overlapping sliding windows. **B.** Phylogenetic tree of ricin-related genes encoding intact RIPs among Euphorbiaceae species. The tree was constructed based on maximum likelihood. **C.** Motif prediction for the RIP homologous family. Five color boxes represent five motifs. **D.** Expression patterns of 8 RIP-encoding genes across different tissues of castor. **E.** Ricinoleic acid synthesis pathway. Expression profiles of the genes involved in the ricinoleic acid synthesis were shown. **F.** Manhattan plots for the hundred-grain weight (left) and seed volume (right) in the full population. The horizontal red line represents the significance threshold (−Log_10_*P* value > 6). The arrow indicates the peak signal containing the newly identified candidate genes. Jat, *Jatropha curcas* L. (physic nut); Ver, *Vernicia fordii* (tung tree); Rub, *Hevea brasiliensis* (rubber tree); RIP, ribosome-inactivating protein; RTA, ricin toxin A chain; RTB, ricin toxin B chain; L, leaf; R, root; S, stem; F, flower; EDS, early seed developmental stage (2–3 weeks after pollination); MDS, middle seed developmental stage (4–7 weeks after pollination); LDS, late seed developmental stage (mature dry seed); DS, dormant seed; *α/β-PDH*, alpha/beta-subunit of pyruvate dehydrogenase; *DHLAT*, dihydrolipoyllysine-residue acetyltransferase; *LPD*, lipoamide dehydrogenase; *BCCP1/2*, biotin carboxyl carrier protein 1/2; *α/β-CT*, alpha/beta-subunit of carboxyltransferase; *BC*, biotin carboxylase; *MCMT*, malonyl CoA-ACP malonyltransferase; *KASII/III*, ketoacyl-ACP synthase II/III; *KAR*, ketoacyl-ACP reductase; *HAD*, 3-hydroxyacyl-ACP dehydrase; *EAR*, enoyl-ACP reductase; *SAD*, stearoyl-ACP desaturase; *LACS*, long-chain acyl-CoA synthetase; *PDAT1/2*, phospholipid diacylglycerol acyltransferase 1/2; *FAH12*, fatty acid hydroxylase 12; *PDCT*, phosphatidylcholine diacylglycerol cholinephosphotransferase; *GPAT6/8/9*, glycerol-3-phosphate acyltransferase 6/8/9; *LPAT*, lysophosphatidyl acyltransferase; *PAP*, phosphatidic acid phosphatase; *DGAT1/2*, diacylglycerol acyltransferase 1/2; *OLE*, oleosin; ACP, acyl carrier protein; PC, phospholipid choline; PA, phosphatidic acid; RR, diricinoleoyl-sn-glycerol; RRR, triricinolein; RRRR, (diricinoleoyl-ricinoleoyl)-diricinoleoyl-glycerol; TAG, triacylglycerol; *UXS6*, uridine diphosphate (UDP)-xylose synthase 6.

The wild castor WT05 and the reference cultivar NSL4733 have a similar genome size, but their assembly qualities are quite distinct. First, the numbers of scaffolds assembled in the WT05 and NSL4733 genomes are 146 and 25,828, respectively. The contig N50 length and scaffold N50 length of the WT05 genome are 425 (8963.1 kb *vs.* 21.1 kb) and 64 (31927.7 kb *vs.* 496.5 kb) times those of the NSL4733 genome, respectively (Table 1). Moreover, based on genome collinearity statistics, only 666 scaffolds (253,067,746 bp in length) in the NSL4733 genome could be completely aligned with 10 chromosomes of WT05, and most of the remaining unaligned scaffolds may be short repetitive sequences (Table S8). These results indicate that the newly assembled castor genome has high sequence homology and chromosome integrity, which greatly improves the quality of the castor genome (Figure 2A, Figures S5 and S6). Additionally, the genome sequence similarity between the two versions was estimated to be 99.16%, suggesting that the two genomes have not diverged much yet (Table S9).

Furthermore, we identified 1,011,145 single nucleotide polymorphisms (SNPs) and 1,197,665 insertions and deletions (indels) in the WT05 genome when compared to the NSL4733 genome, resulting in an average density of 3.20 SNPs and 3.79 indels per kilobase, respectively (Figure S7A–C; Table S10). We also identified six types of structural variations (SVs) in the WT05 genome, including 8.09% inserted duplication (DUP), 0.82% other inserted sequence (BRK), 0.82% rearrangement with another sequence (SEQ), 0.67% gap between two mutually consistent alignments (GAP), 0.06% rearrangement (JMP), and 0.008% rearrangement with inversion (INV) (Figure S7D). These variants provide more targets for further molecule research.

A better genome assembly would allow us to annotate the structure and function of genes more accurately. By comparing gene annotation between the two genomes, we found that the number of genes annotated in the NSL4733 genome is greater than that in the WT05 genome; however, the minimum, maximum, and average lengths of coding sequences (CDSs) in the NSL4733 genome are shorter than those in the WT05 genome (Table S11). This result reflects incomplete gene annotation in the NSL4733 genome, likely caused by the fragmented sequence assembly. For instance, the genes Chr03m1425 and Chr01m0783 in the WT05 genome were annotated as containing 9 and 14 exons, respectively, which was validated by RNA-seq data from 5 castor tissues, whereas in the NSL4733 genome, only 3 and 6 exons were annotated in these two genes, respectively. Detailed examination showed that these two genes are located at the ends of the shorter scaffolds, and thus, the missing exons are the result of an incomplete assembly (Figure S8). Furthermore, in the process of gene annotation of the WT05 genome, large RNA-seq datasets from 17 castor samples were collected for correcting gene annotation. Some truncated genes in the previous NSL4733 version were re-annotated as complete genes in the new annotation. For example, the Chr09m1125 gene contains two short sequences (30064.t000012 and 30064.t000013) in the NSL4733 version; a similar result was obtained for the Chr10m1108 gene (Figure S9). These results indicate that the gene annotation has been vastly improved in the newly obtained WT05 genome, providing accurate genetic information for evolutionary and functional genomic studies on castor.

Take advantage of the newly obtained WT05 genome, we re-annotated two families of important genes in castor, namely, ricin-related genes and genes involved in ricinoleic acid synthesis. First, we identified 25 ricin-related genes, which distribute in 5 scaffolds and encode 8 ribosome-inactivating proteins (RIPs) with both ricin A and B chains, 9 ricin A chain proteins, and 8 ricin B chain proteins (Table S12). Specifically, 22 of the 25 genes are concentrated in 4 segments of chromosome 8 (Figure S10A and B). In contrast, 28 ricin-related genes scatter along 17 scaffolds in NSL4733 assembling. Moreover, two sets of truncated adjacent gene pairs are supposed to derive from two pseudogenes (Figure S10C).

Based on the annotation, we attempted to uncover the mechanism underlying the high toxicity of castor. Ricin has been identified as a type II RIP containing two domains: one is the active domain (ricin toxin A chain, RTA) which removes specific adenine residues from rRNA, and the other is the lectin domain (ricin toxin B chain, RTB) which allows ricin to bind to cell surface and then enter the cell through endocytosis. These two domains are connected by a disulfide bond. Notably, there are 8 copies of ricin-related genes encoding intact RIPs in the WT05 genome, whereas there are 2, 1, and 1 homologous genes found in rubber tree, tung tree, and physic nut, respectively (Figure 2B and C). Further sequence alignment among these homologs revealed that a 43-aa motif located in the middle of RTA chain is highly divergent between castor and other plants without ricin, including rubber tree, tung tree, oil palm, and tea tree (Figure 2C, the pink box; Table S13). Interestingly, the Tyr129 site in this variable motif has previously been identified as one of the key active sites in RTA that is involved in the depurination of a specific residue from the 28S rRNA, and its mutation is able to result in a seven-fold decrease in enzyme activity [15] (Figure S11). These results suggest that this 43-aa motif in RTA plays a critical role in the action of ricin. Furthermore, we investigated the expression profiles of the ricin-related genes by integrating RNA-seq data from different castor tissues, which showed a tissue-specific expression pattern. Among the eight RIP-encoding genes, Chr08m1661, Chr08m1663, Chr08m1667, and Chr08m1657 are specifically and highly expressed in seeds at different developmental stages, and Chr08m1621 is mainly expressed in leaves and stems (Figure 2D). The RIP-encoding genes show clearly higher transcriptional activity in seeds than in other tissues, consistent with the observation that castor seeds have higher toxicity than other tissues. In contrast, the genes encoding only ricin A or B chain prefer to have relatively low or no expression across the tissues (Figure S12A and B). Due to the lack of some conserved motifs in the proteins encoded by these genes, it is not clear whether they still have RIP function. These comprehensive expression profiles provide a good reference for functional research of ricin-related genes.

On the other hand, we annotated 301 genes putatively related to fatty acid synthesis and reconstructed the ricinoleic acid synthesis pathway (Figure S13; Table S14). We diagrammed the fatty acid synthesis pathway with the corresponding genes involved in ricinoleic acid synthesis, and integrated transcriptome to identify key genes showing differential transcript abundance across different tissues and seed developmental stages of WT05 castor (Figure 2E). In detail, several genes, including acetyl-CoA carboxylase genes [biotin carboxyl carrier protein 1 (*BCCP1*), *BCCP2*, alpha-subunit of carboxyltransferase (*α-CT*), beta-subunit of carboxyltransferase (*β-CT*), and biotin carboxylase (*BC*)], malonyl CoA-ACP malonyltransferase (*MCMT*), enoyl-ACP reductase (*EAR*; RcuChr10m2241), beta-ketoacyl-ACP synthase II (*KASII*), stearoyl-ACP desaturase (*SAD*; RcuChr02m1181 and RcuChr07m3084), phospholipid:diacylglycerol acyltransferase (*PDAT*), oleosin (*OLE*), and fatty acid hydroxylase 12 (*FAH12*), were relatively highly expressed in the seeds compared with in the roots, stems, leaves, and flowers, which is consistent with the enrichment of ricinoleic acid in castor seeds (Figure 2E). Specifically, in the pathway of ricinoleic acid synthesis, we found that four key genes, *BCCP2*, *EAR*, *SAD*, and *FAH12*, showed relatively higher expression in the early and middle seed developmental stage (EDS and MDS) and decreased expression in the late seed developmental stage (LDS), followed by no or weak expression in the stage of dormancy (DS). This expression trend is consistent with the accumulation of fatty acids in castor seeds [16].

The genome assembly and gene annotation in WT05 greatly improve the quality of the reference genome of castor, which allows us to identify genetic variations and perform GWAS analysis more accurately. Taking advantage of the newly obtained WT05 genome, we reanalyzed the resequencing data from 385 Chinese castor lines that have been published in 2019 [17]. 75 SNP sites were randomly selected for validation by Sanger sequencing, and 99.72% (1421/1425) of SNP sites were correctly detected in 19 samples (Table S15). We totally identified 2218 SNPs that are significantly correlated to 9 agricultural traits (*P* < 1.0 × 10^−6^), of which 602 SNPs were not able to be identified in a previous analysis [17]. This GWAS analysis not only validated a great many of the known controlling loci but also annotated lots of new candidate markers associated with agricultural traits that were unable to be detected in the previous analysis (Figure 2F, Figure S14). For example, we detected one novel signal in chromosome 3, in which 44 SNPs are significantly associated with hundred-grain weight. These SNPs are located in the upstream 3.25–17.6 kb region (scattered in Chr03:2564–2565.6 kb) of the LOC107262598 gene that was annotated as a homolog of the *RICESLEEPER2* gene in rice. *RICESLEEPER2* has been reported to be associated with the number of seeds, and its mutant trends to produce empty panicles, resulting in very few seeds in rice [18]. Another new signal was detected in chromosome 8, in which 3 SNPs are significantly associated with seed volume and located in the upstream 1.6–1.8 kb region of the LOC8281893 gene. The rice homolog of this gene encodes UDP-glucuronic acid decarboxylase (OsUXS) and plays an important role in a certain stage of rice seed development [19]. More novel SNPs associated with the 9 agricultural traits are listed in Table S16. Therefore, the WT05 genome provides a high-quality reference for population genetics research and molecular breeding of castor.

### Gene family expansion associated with photosynthesis

**Figure 3:**
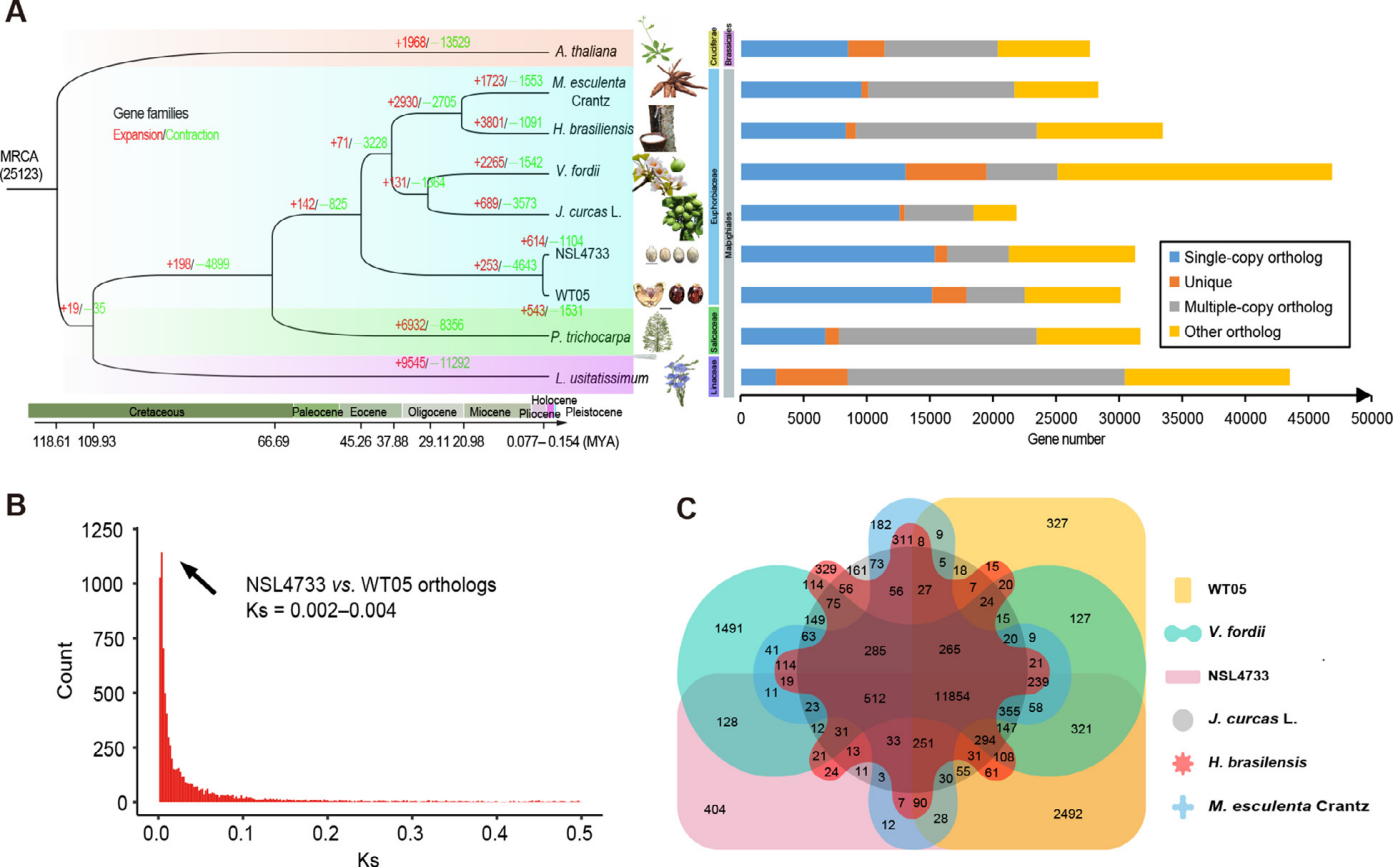
**Evolutionary analyses of the WT05 genome compared with the genomes of other Euphorbiaceae plants** **A.** Phylogenetic relationships and divergence times between wild castor and other Euphorbiaceae species. *A. thaliana*, *L. usitatissimum*, and *P. trichocarpa* were used as outgroups **B.** Distribution of the synonymous substitution rate (Ks) between WT05 and NSL4733. **C.** Venn diagram showing the gene families in six Euphorbiaceae species. The numbers indicate gene families identified among all selected species. MYA, million years ago; *A. thaliana*, *Arabidopsis thaliana*; *M. esculenta* Crantz, *Manihot esculenta* Crantz; *H. brasiliensis*, *Hevea brasiliensis*; *V. fordii*, *Vernicia fordii*; *J. curcas* L., *Jatropha curcas* L.; *L. usitatissimum*, *Linum usitatissimum*; *P. trichocarpa*, *Populus trichocarpa*.

To investigate the phylogenetic position of castor in Euphorbiaceae species, especially the divergence time between wild and cultivated castors, we constructed a phylogenetic tree for five Euphorbiaceae species, including *R. communis* L. (WT05 and NSL4733), *M. esculenta* Crantz, *J. curcas* L., *H. brasiliensis*, and *V. fordii*, with *Arabidopsis thaliana*, *Linum usitatissimum*, and *P. trichocarpa* as outgroups, using 622 single-copy gene families. As expected, the wild castor is most closely related to cultivated castor (Figure 3A), and the tree topology is consistent with previous research [20]. To estimate the divergence time between wild and cultivated castors, we used the 10,906 collinear genes from a total of 722 syntenic blocks between two genomes to calculate the synonymous substitution rate (Ks) distribution, and the results showed peaks at 0.002 to 0.004. According to the substitution rate of 1.3 × 10^−8^ mutations per locus per year, we estimated the divergence time to be 0.077–0.154 MYA (Figure 3B). The divergence time was also predicted by the McMctree program based on the phylogenetic tree, which was estimated to be 1.16 MYA. Since both of these divergence times are much earlier than the cultivation time (~ 1000 years ago) of castor, we speculated that the wild castor WT05 is not a direct ancestor of the cultivar NSL4733.

To understand the potential adaptive mechanisms of wild castor growing in harsh conditions, such as intense UV radiation, high light, and drought, we first performed gene family expansion analysis since specific gene family expansion or contraction often corresponds to the adaptive evolution of species. Based on the species in the phylogenetic tree, we identified 25,123 gene families. Among these gene families, 11,854 were shared by the five species (*R. communis* L., *M. esculenta* Crantz, *J. curcas* L., *H. brasiliensis*, and *V. fordii*) of the Euphorbiaceae family (Figure 3C). Through comparison between the wild and cultivated castors, we totally identified 147 gene families that were significantly expanded (*P* < 0.01; Table S17) and 254 gene families that were significantly contracted (*P* < 0.01). Gene Ontology (GO) annotations revealed that the functions of the extended families were significantly enriched in photosynthesis and light responses. The specific enriched pathways included the biological processes of photosynthesis, light reaction (*P* = 1.55E−05), photosynthetic electron transport (*P* = 1.05E−05), photosynthesis (*P* = 3.43E−03), and response to oxidative stress (*P* = 4.55E−07), the molecular functions of chlorophyll binding (*P* = 2.01E−06) and peroxidase activity (*P* = 1.74E−05), and the cellular components of photosystem (*P* = 8.81E−04), photosynthetic membrane (*P* = 1.31E−03), and thylakoid (*P* = 1.36E−03) (Table S18). As an example, one significantly expanded gene family, photosystem II reaction center protein B (*PSBB*) [21], which is involved in photosynthesis, light reaction, and photosynthetic electron transport in photosystem I, contains four copies in the wild genome but has only two copies in the cultivated genome (Figure S15A). A similar result was found when we compared the gene families of castor with those of other four Euphorbiaceae species as well as those from *A*. *thaliana*, *L*. *usitatissimum*, and *P. trichocarpa*. 16 gene families containing 95 genes were significantly expanded in the wild castor genome (*P* < 0.05), one of which contains 4 genes involved in photosynthesis (Tables S19 and S20). We also verified the accuracy of the copy number amplification events of the *PSBB* gene family by alignment of transcriptomes from different tissues of castor (Figure S15B). These results suggest that the expansion of photosynthesis-related genes in wild castor could be potentially associated with the adaptation to intense light in the desert region.

### Positive selection associated with DNA repair

**Figure 4:**
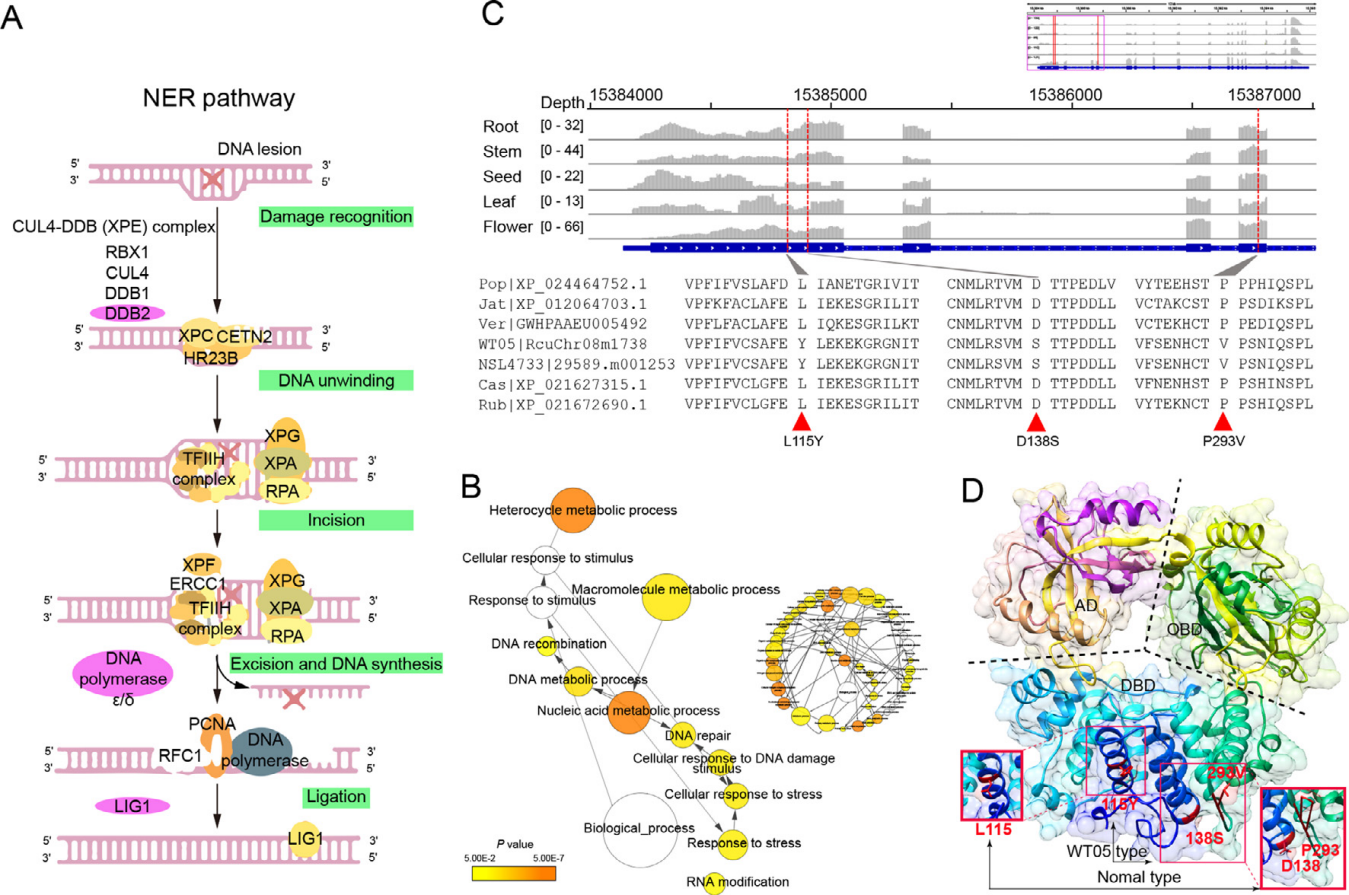
**PSGs involved in DNA repair** **A.** Key genes play roles in the NER pathway. **B.** GO enrichment of PSGs. The circle size is proportional to the number of genes in each category, and the colors are related to *P* values for the statistical significance of the enrichment. Relative positions were revised manually to reduce the complexity of the image. **C.** Amino acid sequence alignment of the LIG1 proteins from castor (WT05 and NSL4733) and other Euphorbiaceae species as well as *P*. *trichocarpa*. The upper panel shows the gene structure and expression abundance of *LIG1* across five tissues of WT05 castor. The gray columns represent the transcriptome alignment depth, and the dotted red lines indicate the positions of allelic mutations. **D.** 3D structure simulation of the castor LIG1 protein. PSG, positively selected gene; NER, nucleotide excision repair; CUL4, cullin 4; DDB, damaged DNA-binding protein; RBX1, ring-box 1; XPE, xeroderma pigmentosum xeroderma complementation group E; XPC, xeroderma pigmentosum complementation group C; CETN2, centrin-2; HR23B, RAD23 homolog B; XPG, xeroderma pigmentosum xeroderma complementation group G; XPA, xeroderma pigmentosum xeroderma complementation group A; RPA, replication protein A1; XPF, xeroderma pigmentosum xeroderma complementation group F; ERCC1, excision repair cross-complementation group 1; TFIIH, transcription factor IIH; PCNA, proliferating cell nuclear antigen; RFC1, replication factor C subunit 1; LIG1, DNA ligase 1; Pop, *P. trichocarpa*; Cas, *M. esculenta* Crantz; DBD, DNA-binding domain; AD, adenylation domain; OBD, OB-fold domain.

Sunlight is essential for plant growth and constantly replenishes energy through photosynthesis; thus, plants cannot survive without light. However, wild castor plants grow in the desert region on the African Plateau, so they must tolerate ultrastrong UV radiation, which inevitably causes DNA lesions to varying degrees. Considering the impact of intense UV radiation or high light intensity in tropical desert areas on DNA damage, it is hypothesized that wild castor has developed strong DNA repair systems to adapt to intense UV radiation during long-term evolution [22], [23]. Under natural selection, advantageous mutations are usually fixed in the population during adaptive evolution. To identify potential genetic variations associated with the DNA repair pathways in wild castor, we performed positive selection analysis of 3024 single-copy homologous genes among wild and cultivated castors as well as other four Euphorbiaceae species using the branch-site model of the PAML package. As a result, 476 significant positively selected genes (PSGs) were identified in the WT05 genome (*ω* > 1, *P* < 0.05). Kyoto Encyclopedia of Genes and Genomes (KEGG) functional classification of these 476 PSGs showed that several categories associated with base excision repair (BER), purine/pyrimidine metabolism, non-homologous end-joining (NHEJ), nucleotide excision repair (NER), homologous recombination (HR), DNA replication, and mismatch repair (MR) were enriched (Table S21). GO enrichment analysis also revealed that these PSGs were enriched in several categories associated with DNA repair, cellular response to DNA damage stimulus, response to stress, and cellular response to stimulus (Figure 4A and B; Table S22). These results suggest that there are indeed many genes related to DNA repair undergoing positive selection during the long-term adaptive evolution of the wild castor genome. Similarly to those observed in *Crucihimalaya himalaica* [24]*,* Tibetan antelope [25], Tibetan chickens [26], and ectothermic snakes [27], some genes responsive to DNA damage and repair were also been identified under positive selection pressure in order to adapt to high altitude environment. These results consistently suggest that the evolution of DNA repair system is an important common mechanism for organisms to adapt to extreme environments.

Here, we identified 21 PSGs associated with DNA repair and light response, 9 of which play key roles in DNA repair pathways, including NER, BER, and MR (Table S23). For example, the DNA ligase 1 (*LIG1*) gene acts a pivotal part in both DNA replication and excision repair pathways, which could repair both single- and double-strand break lesions [28]. Three amino acid substitutions (L115Y, D138S, and P293V) were identified in the LIG1 protein of both wild and cultivated castors when compared to other four Euphorbiaceae species and *P. trichocarpa*, which was also confirmed by transcriptome data from roots, stems, leaves, seeds, and flowers of the castor (Figure 4C). To explore whether these substitutions are located in protein domains, we further simulated the protein three-dimensional (3D) structure to examine the possible effects of the mutations on the enzyme structure using Phyre2 [29]. As a result, 616 residues (77% of the protein sequence) were modeled with 100% confidence based on the single highest scoring template, and the structure was similar to the crystal structure of human LIG1 [30]. We found that all three amino acid substitutions are located in the DNA-binding domain (DBD) (Figure 4D). A previous study has demonstrated that chemical- and radiation-induced allelic mutations in the DBD region impair DNA repair pathways by decreasing enzymatic activities [31]. In addition to *LIG1*, damaged DNA-binding protein 2 (*DDB2*; RcuChr01m3516) also plays a synergistic role in the excision repair process, which can maintain genome integrity under UV exposure in *A. thaliana* [32] and even in mammals [33]. The DNA polymerase gene RcuChr01m0783 encodes the homolog of mammalian DNA polymerase lambda, which is involved in repairing UV-B-induced DNA damage [34]. Furthermore, RcuChr07m1522 encodes a homolog of the UV-B photoreceptor UVB-RESISTANCE8 (UVR8), which is involved in response to UV-B radiation and induces photomorphogenic responses, such as UV-B acclimation and tolerance [35]; RcuChr05m1993 encodes a homolog of the RECG1 DNA translocase, which has been reported as a key factor involved in the process of mitochondrial DNA recombination monitoring, repair, and segregation in *A. thaliana* [36]. Of these genes, DNA polymerase gene involved in the pathways of NER has also been identified in *C. himalaica* (Table S24). NER, BER, and MR are particularly important excision mechanisms that eliminate DNA damage caused by UV radiation and any other stressors [23]. These results suggest that positive selection of genes related to DNA repair pathways in wild castor may be a potential defense mechanism for adaptation to UV or intense high light exposure.

Other abiotic stresses such as high temperature, drought, and high salinity are also typical features in tropical desert areas. Here, we identified a group of PSGs that are potentially involved in stress responses (Table S25). First, we identified RcuChr03m1916 encoding a homolog of *A. thaliana* heat shock transcription factor A2 (AtHsfA2). Up-regulation of *AtHsfA2* tends to improve heat tolerance in *A. thaliana* [37]. Its homolog *AtHsfA1* has been reported to confer resistance to heat stress [38]. Another gene, RcuChr02m0839, encodes a homolog of the *A. thaliana* chaperone protein AtDjB1, which belongs to the DnaJ heat shock protein family and participates in osmotic stress tolerance through ABA signaling regulatory pathways [39]. Additionally, RcuChr06m2064 encodes a zinc finger protein whose homologs have been reported to play a functional role in salt tolerance in rice [40], *A. thaliana* [41], cotton [42], and poplar [43]; RcuChr10m1484 and RcuChr05m0192 encode a homolog of *A. thaliana* drought-induced protein19 (AtDi19) [44] and *Zea mays* early responsive to dehydration 4 (ZmERD4) [45], respectively, which are correlated with drought resistance response. These results suggest that the genetic variations in these PGSs could be closely associated with environmental adaptability.

### Selection signals in the wild castor population

**Figure 5:**
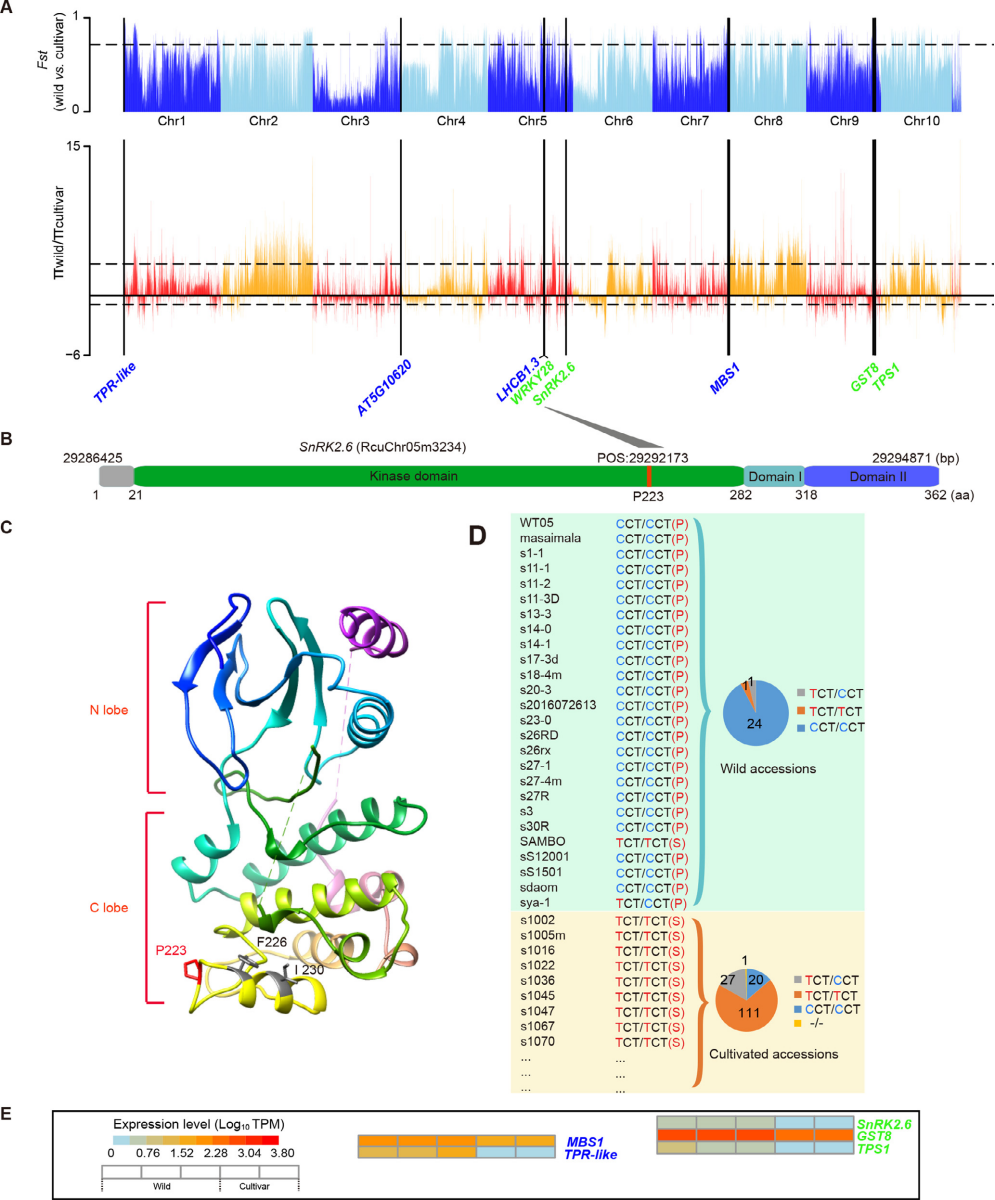
**Genomic diversity comparison between wild and cultivated castor varieties** **A.** Bar plots of the *Fst* (upper) and π_wild_/π_cultivar_ (bottom) values for the whole genome between wild and cultivated castor varieties. The horizontal dotted black lines indicate the top 10% selection threshold, and the vertical solid black lines indicate the locations of the genes identified to be involved in drought responses (in green) and strong light responses (in blue) in the selection window (10-kb non-overlapping sliding window). **B.** Sequence characteristics of the SnRK2.6 protein. The red line indicates the position of the allelic variant. **C.** 3D structure model of SnRK2.6. The allelic variant is highlighted in red, and two adjacent sites are highlighted in gray. **D.** Allelic information for sequence variants of the *SnRK2.6* gene among wild and cultivated castors. The pie charts show the allele frequencies of the causal polymorphisms for the *SnRK2.6* gene in different wild and cultivated castor varieties. The numbers in the pie chart represent the number of allele variations at position 29292173 (P223S) of chromosome 5 in the wild (total 26) or cultivated (total 159) population. Here, only a portion of the cultivated castor samples are shown (more detailed information is provided in Table S29). **E.** Comparison of the expression levels of genes undergoing selective pressure between wild and cultivated castor leaves. “-/-” indicates allele missing.

The wild castor population is growing under the strong pressure of natural selection in the tropical desert area. Consequently, some genomic regions or genes associated with environmental adaptation in the wild castor population are expected to evolve with high conservation under natural selection pressure. Based on this principle, we calculated the ratio of genetic diversity (*π*_wild_/*π*_cultivar_) and population differentiation (*Fst*) between 26 wild germplasms and 159 cultivated germplasms in a non-overlapping window of 10 kb (Table S26). Setting the selection threshold to top 10% of the *Fst* values and top 10% of the *π*_wild_/*π*_cultivar_ values, 1132 genomic windows were identified to be associated with selected signals (Figure S16; Table S27). Functional analysis of the genes located in these selected regions identified four genes involved in drought responses and four genes involved in strong light responses (Figure 5A; Tables S28 and S29). These results suggest that some genes related to environmental stresses have undergone natural selection during the evolution of wild castor.

In the tropical desert region, wild castor is exposed to not only UV radiation but also drought stress. Among the four genes (RcuChr05m3234, RcuChr09m2155, RcuChr09m2100, and RcuChr05m2127) identified to be involved in drought responses, RcuChr05m3234 encodes a homolog of *A. thaliana* sucrose nonfermenting 1-related protein kinase 2.6 (SnRK2.6) [46]. The SnRK2 protein family, as a protein kinase family, plays important roles in the activation of stress response signals, such as signals associated with the response to salt, drought, and osmotic stress [47]. Our comparative genomics analyses found that there are totally 46 SNPs around in the RcuChr05m3234 gene. Among them, 1) only two SNPs are located in the exonic region: one is non-synonymous (SNP: 29292173; C > T) which causes an amino acid change from phenylalanine to serine (P223S) (Figure 5B), and the other is synonymous; 2) one is in the upstream regulatory region; 3) five are in the downstream regulatory region, and 4) others are located in 5′-UTR (9), 3′-UTR (10), and intronic (19) regions. Based on the simulated 3D structural model, we found that P223S is located within the kinase domain of SnRK2.6, a structural motif on the C lobe of SnRK2.6 adjacent to the potential location of the activation loop (Figure 5C). Previous research has suggested that mutations of the two adjacent sites F226D and I230D can result in complete dissociation between SnRK2.6 and abscisic acid insensitive 1 (ABI1) in *A. thaliana* [48]. Furthermore, we found that the number of allelic variations at position 29292173 (P223S) of chromosome 5 is obviously different between wild and cultivated populations. Among the 26 wild accessions, 24 carry homozygous C alleles. However, among the 159 cultivated accessions, 111 carry homozygous T alleles, and the rest are 27 heterozygotes, 20 homozygotes carrying C alleles, and 1 missing this allele (Figure 5D). Moreover, *SnRK2.6* showed higher expression in wild castor than in cultivated castor (Figure 5E). Another gene, RcuChr09m2155, encodes a homolog of trehalose-6-phosphate synthase (TPS1), which is involved in trehalose biosynthesis. It has been identified as a drought-resistant gene in drought-tolerant cassava, physic nut, and castor crops. In cassava, previous research has shown that higher amounts of trehalose contribute to higher drought stress tolerance [49]. In rice, a study has also demonstrated that overexpression of *OsTPS1* enhances tolerance to abiotic stresses, including cold, high-salinity, and drought stress. Three non-synonymous SNPs are located in the exon region of RcuChr09m2155, causing corresponding amino acid substitutions. Further analysis revealed that all wild accessions in our collection carry homozygous C, A, and A alleles at positions 25967789, 25945030, and 25941524 of chromosome 9, respectively; however, 89.94% (143/159), 91.19% (145/159), and 89.94% (143/159) of the cultivated accessions carry homozygous T/C/G alleles or heterozygous alleles at corresponding SNP sites (Figure S17). Expression profiles revealed that *TPS1* in wild castor leaves had a higher expression level than that in cultivated castor leaves (Figure 5E). Additionally, RcuChr09m2100 and RcuChr05m2127, encode a homolog of the glutathione transferase *GST8* [50] and transcriptional factor *WRKY28* [51] in *Arabidopsis*, respectively, both of which have been suggested to play potential roles in drought and salt stress responses in *Arabidopsis*. Genetic variations were found in UTRs or intronic regions of these two genes, and the expression level of *GST8* was higher in wild castor leaves than in cultivated castor leaves (Figure 5E).

For the four genes identified to be involved in strong light responses, RcuChr01m0050, RcuChr03m2352, RcuChr05m2118, and RcuChr07m3025 separately encode a homolog of a tetratricopeptide repeat (TPR)-like protein [52], a light-sensing related methyltransferase protein (encoded by AT5G10620 in *A. thaliana*) [53], light-harvesting chlorophyll A/B binding protein 1.3 (LHCB1.3) [54], and methylene blue sensitivity 1 (MBS1) [55]. The function of *MBS1* has been well studied. Knockout of *MBS1* in *Arabidopsis* results in hypersensitivity to photooxidative stress, whereas overexpression leads to tolerance to intense light [55]. Evidence has also proved that MBS1 couples with β-cyclocitral to induce transport of singlet oxygen (^1^O_2_) to the nucleus, ultimately leading to photoacclimation [56]. Expression profiles showed that the expression levels of *TPR-like* and *MBS1* were relatively higher in wild castor leaves than in cultivated castor leaves. These results reflect a microcosm of the adaptive evolution of castor in arid, high-light tropical deserts.

## Discussion

Wild castor plants growing in tropical desert regions of the African Plateau are exposed to a variety of abiotic and biotic stresses under harsh environmental conditions, such as drought, salinity, and, especially, UV damage. To adapt to unique conditions, wild castor has developed a series of self-defense systems that provide valuable germplasm resources with advantageous characteristics, such as resistance to UV damage and drought. These traits are highly valued in castor breeding. In this study, we assembled one chromosome-level genome of wild castor, providing a high-quality reference for genomic studies of castor. Furthermore, through comparative genomic analyses with cultivated castor and other four Euphorbiaceae plants, we revealed that a great number of genes associated with stress responses, especially in responses to UV-induced DNA damage and repair, have undergone positive selection and harbor many advantageous variations for castor improvement.

The wild castor WT05 genome was assembled at the chromosome level with high consistency and integrity, greatly improving the quality of the reference genome for castor. Moreover, based on the completeness of the WT05 genome, the gene structure in this castor genome was annotated more precisely than that in the previous NSL4733 version. Additionally, we performed careful gene functional annotation and characterized two classes of important genes in castor, including ricin-related genes and genes associated with fatty acid biosynthesis. Taking advantage of the WT05 genome, we identified genetic variations based on the resequencing data of 385 Chinese castor lines that have been published in 2019 and performed GWAS analysis with 9 agricultural traits. We detected novel SNPs significantly associated with the 9 agricultural traits, which were not able to be found in the previous study. All of these results confirmed that the WT05 genome version is markedly improved over the previous version, thus providing a better reference for studies on castor.

The intense UV radiation environment frequently leads to DNA damage by inducing nucleotide structure lesions such as intra/inter-strand cross-links, cleavage of phosphodiester bonds, and single/double-strand DNA breaks, which inevitably cause errors in transcription or translation, probably resulting in highly cytotoxic lesions and even potentially lethal lesions [23], [57]. Via consistent efforts made in previous studies, DNA repair mechanisms have been well characterized, including the mechanisms of photoreactivation, excision repair, DNA polymerase activity, mutagenic repair, and lesion bypass, as well as recombinational repair [58], [59]. In natural environments, the genomes of species change constantly in response to UV damage or other stresses. The following are some typical examples that have been well identified. In rice, modification of amino acid residues in the cyclobutane pyrimidine dimer (CPD) photolyase appears to decrease its activity in response to UV radiation [60]. SNP variation in *Pinus yunnanensis* occurs in response to UV radiation at high altitudes [61]. The cyanobacterium *Trichormus* sp. growing on the Qinghai-Tibet Plateau (QTP) develops UV-absorbing mycosporine-like amino acids in order to defend against UV radiation, and the UV resistance gene encoding O-methyltransferase undergoes positive selection [62]. In yeast, specific single-amino-acid changes in the different loci of histone H4 enhance UV tolerance and DNA repair [63]. Currently, as the ozone layer thins, intense UV radiation is particularly important as an environmental stress factor [64]. A better understanding of plant DNA repair processes will help accelerate genome engineering through traditional and targeted approaches to address the heightened changes in the environment.

For castor, through evolutionary and comparative analyses, we found that a great proportion of genes involved in DNA repair pathways, including NER, BER, MR, double-strand break repair (DBR), and HR, have undergone positive selection and gene family expansion, such as *LIG1* which encodes a DNA ligase functioning in NER, BER, and MR. This probably reflects the adaptation of wild castor to intense UV radiation in the tropical desert of Africa. Coincidentally, it has been reported that PSGs are enriched in the DNA repair pathways in some species that live under intense UV radiation, such as the alpine plant *Cushion willow* [65] and the high-altitude plants *Crucihimalaya himalaica* [24], Tibetan highland barley [66], Tibetan hot-spring snake [27], Tibetan antelope [25], and Tibetan chicken [26]. These results suggest that species growing in tropical deserts or high elevation areas with intense UV radiation usually develop a self-protection and defense system against harsh environmental stresses over the course of long-term evolution.

Additionally, a number of genes related to stress responses also undergo gene family expansion or positive selection in the wild castor genome, including some key genes involved in drought responses, such as *SnRK2.6*, *GST8*, and *TPS1*. These results provide novel insights into the molecular mechanisms underlying the adaptation of wild castor to abiotic stresses and provide a set of genes and alleles as potential targets for castor improvement.

In summary, we assembled a chromosome-level genome of wild castor, providing high-quality and precise reference sequence and gene annotation for evolutionary and functional genomic studies on castor. Moreover, our results reveal the genetic basis underlying the mechanism of adaptation of wild castor to extreme conditions, including intense UV radiation and drought, providing a foundation for understanding the adaptive strategies of plants to harsh environments. The identification of the genes under positive selection provides a set of potential molecular targets for castor breeding and improvement.

## Materials and methods

### Plant materials

Twenty-six wild castor accessions, as the wild group, were initially collected from Africa [17] (Kenya and Ethiopia). A particular wild castor strain (WT05), which was found specifically at an altitude of more than 2000 m in the semiarid desert region of Kenya, Africa, had the largest seeds and tallest plants, and exhibited strong adaptability to the desert environment, was selected as the material for assembling the wild castor genome.

### Genome sequencing

Wild castor WT05 collected from Kenya, Africa was cultivated in the Wuhan botanical garden, Wuhan, China. The sampling details were as follows. Young fresh leaves were first harvested and deposited in liquid nitrogen for genomic DNA extraction. Then, high-quality genomic DNA was extracted using Plant Genomic DNA Kit (Qiagen, San Diego, CA). The extracted high-quality genomic DNA was divided into two parts, one for short-read sequencing on the Illumina NovaSeq 6000 platform and the other for long-read sequencing on the GridION X5 platform with libraries of 20 kb insert size based on Oxford Nanopore technology. We also sampled the RNA-seq materials from the leaves, roots, seeds, and stems of wild castor and extracted total RNA using the QIAGEN RNeasy Plant Mini Kit (QIAGEN, Hilden, Germany). In addition, samples for Hi-C library construction were collected from the same plants and sequenced through the Illumina HiSeq platform.

### Genome assembly

We performed genome assembly with a combination of long Nanopore reads, Illumina short reads, and Hi-C sequencing data. Sequence corrections were performed using Canu (v1.7) [67] with default parameters. Corrected sequences were assembled using SMARTdenovo (https://github.com/ruanjue/smartdenovo) with default parameters (Table S30). Then, the assembled genome was corrected by nanopolish with parameters (-t 4 --min-candidate-frequency 0.05) (https://github.com/jts/nanopolish.git, v0.9.2) using the long-read sequences and polished (five rounds) by pilon (v1.21) using the short-read sequences to finally generate high-quality consensus contigs with default parameters (Figure S18). Finally, Hi-C data help to anchor contigs into ten chromosome-level scaffolds base on the 3D-DNA program (v180922) [68] with the parameters “-r 2 --mode haploid” and the Juicer pipeline (v1.5.7) [69] with the parameters “-s DpnII”. Then, juicerbox was used for genome visualization and manual correction.

### Genome size and heterozygosity estimation

We estimated the genome size of WT05 based on the k-mer method using the Illumina short-read sequences. Quality-filtered reads were used for 25-mer frequency distribution analysis according to the Jellyfish program (v1.1.10) [70] with the parameters “-m 25 -s 350M”. The heterozygosity rate of the WT05 genome was calculated by GenomeScope software (v1.0) [71] with the parameter “k = 25”.

### Gene prediction and annotation

Three pieces of evidence from homology comparison, *de novo* prediction, and transcriptome-based analyses were combined for gene prediction. First, for homology-based comparison, we downloaded protein sequences of eight species, including cottonwood (*P. trichocarpa*), flax (*L. usitatissimum*), cassava (*M. esculenta* Crantz), a reference version of cultivated castor NSL4733 (*R. communis* L.), physic nut (*J. curcas* L.), rubber tree (*H. brasiliensis*), tung tree (*V. fordii*), and *A. thaliana*. All the protein sequences were mapped to the WT05 draft genome using geneblastA (v1.0.1) with the parameter “-evalue ≤ 1E-5”, and only the best alignment with the highest score was retained for further gene coding region prediction using GeneWise (https://www.ebi.ac.uk/Tools/psa/genewise, v2.2.3) [72]. Second, for *de novo* prediction, we first randomly selected 3000 full-length gene models to train the model and then used Augustus (v3.3.2) [73], Genescan (http://genes.mit.edu/GENSCAN.html, v1.0), and SNAP (http://korflab.ucdavis.edu/software.html, v2013-02-16) [74] with default parameters to predict gene models based on the training set. Third, for transcriptome-based analysis, RNA-seq reads were filtered and trimmed using Trimmomatic (v0.36) [75] with the parameters “LEADING:3 TRAILING:3 SLIDINGWINDOW:4:15 MINLEN:80”. Trimmed reads were mapped to the draft genome using tophat2 (v2.0.12) [76], and then, transcripts were constructed using cufflinks (v2.2.1) [77] and cuffmerge. Open reading frames (ORFs) were predicted by the transdecoder using transcript data and Rfam databases. Finally, gene models from the homology-, *de novo*-, and RNA sequence-based methods were integrated by EvidenceModeler (http://evidencemodeler.github.io/; parameters: --segmentSize 5000000 --overlapSize 10000) and then further updated by PASA [78] (parameters: -c alignAssembly.config -C -R --ALIGNERS blat --TDN tdn.accs --ALT_SPLICE) to generate UTRs and alternative splicing variants. The annotation process refers to the RGAAT pipeline [79].

Gene functions were annotated based on the NR, TrEMBL, and SwissProt (http://web.expasy.org/docs/swiss-prot_guideline.html) [80] databases using Blastp [81] with a threshold of -evalue ≤ 1E−5. Only genes with the best match and highest score were retained. Gene motifs and functional domains were annotated using InterProScan [82]. GO term (http://www.geneontology.org/page/go-database) annotations for genes were available from the INTERPRO and PFAM databases.

Besides, tRNAscan-SE (v1.3.1) with default parameters was used for tRNA annotation. Prediction of rRNAs was conducted by RNAmmer software (v1.2). The non-coding RNAs were identified by employing INFERNAL software to search against the Rfam database.

### Detection and analysis of LTR-RTs

The masking of the repeat sequences was conducted based on homology-based and *de novo* strategies. First, the *de novo* repeat library was constructed by RepeatModeler (http://www.repeatmasker.org/RepeatModeler.html, version open-1.0.8). Then run RepeatMasker (http://www.repeatmasker.org, v1.332) [83] with *de novo* data, and Dfam_Consensus-20181026 and RepBase (v20170127) [84] were used as the query libraries to classify the repeat type. LTR insertion time was calculated by LTR_harvest with the parameters “-similar 90 -vic 10 -seed 20 -seqids yes -minlenltr 100 -maxlenltr 7000 -mintsd 4 -maxtsd 6 -motif TGCA -motifmis 1” and LTR_FINDER software with the parameters “-D 15000 -d 1000 -L 7000 -l 100 -p 20 -C -M 0.9” (http://tlife.fudan.edu.cn/ltr_finder/, v1.06) [85]. Then, run LTR_retriever software (https://github.com/oushujun/LTR_retriever) with default parameters to calculate LTR insertion time. The final results were integrated from the abovementioned results of the three pipelines (LTR_harvest, LTR_FINDER, and LTR_retriever).

### Evaluation of assembly quality

BUSCO (v3) [10] was used to assess the assembly completeness of the new genome. Illumina paired-end reads were used to align to the genome by BWA with default parameters. MCscanX [86] with default parameters was used to identify collinearity blocks. Delta-filter instated in MUMmer package (v3.23) with the parameters “-i 95 -l 1000” was used to filter short sequences less than 1 kb and reserve sequences with identify > 95%. Dnadiff installed in MUMmer package (v3.23) [87] was used to calculate alignment ratio and sequence identity at scaffold level.

### Gene family expansion and contraction

The OrthoMCL package (v2.0.9) [88] was used to identify the orthologous genes among *R. communis* L. (WT05 and NSL4733), *P. trichocarpa*, *L. usitatissimum*, *M. esculenta Crantz*, *J. curcas* L., *H. brasiliensis*, *V. fordii*, and *A. thaliana*. CAFÉ software (v4.1) [89] was used to analyze the expansion and contraction of homologous gene families.

Each significantly expanded and contracted gene family was defined by comparing the cluster size, and *P* < 0.05 was considered significant. GO enrichment analysis of genes was performed using the BiNGO application installed in Cytoscape software (v3.7.2) [90]. The online version of KOBAS software (http://kobas.cbi.pku.edu.cn/index.php, v3.0) [91] was used to find the genes in KEGG pathways that are significantly enriched.

### Evolutionary analysis

Single-copy orthologous genes were used for phylogenetic tree construction through running RAxML software (v8.2.12) [92] with the parameters “-n ex -f a -N 100 -m PROTGAMMAAUTO”, where *A. thaliana*, *P. trichocarpa*, and *L. usitatissimum* were designated as outgroups. The MAFFT software (v7.305b) [93] with default parameters was used to perform multi-protein sequence alignment for each group of single-copy homologous genes; then the protein sequence alignment was converted into codon alignment. The time of species divergence was estimated by the McMctree program. We calculated the synonymous Ks using KaKs_calculator with the NG model. The divergence time between wild and cultivated castors was estimated using the formula T = Ks/2r (r = 1.3 × 10^−8^ per site and per year) [94].

### Prediction of protein structure

Phyre2 [29] (http://www.sbg.bio.ic.ac.uk/phyre2/) was used to predict the protein structure according to the amino acid sequence. Visualization and mutation identification were performed using the Chrmera1.14 software [95]. The motif-based sequence analysis tool MEME Suite (http://alternate.meme-suite.org/meme_5.0.3/doc/meme-format.html, v5.0.3) was used to predict protein sequence motifs.

### Transcript analysis

For transcriptome data, a total of 67 Gb of RNA-seq reads were extracted from 17 samples from different tissues and various developmental stages of different castor varieties (Table S31). Data from 13 samples (a total of 40 Gb) were downloaded from the NCBI SRA database, and data from other 4 samples (approximately 32.5 Gb) were from leaves, stems, roots, and seeds of WT05 cultivated in Wuhan botanical garden, Wuhan, China. High-quality RNA was extracted and then sequenced on the HiSeq 2500 platform. We filtered the low-quality reads by Trimmomatic-0.36 [75], and the parameters were set as LEADING:3 TRAILING:3 SLIDINGWINDOW:4:15 MINLEN:80. Stringtie (v1.3.3) [96] was used to compute the transcript expression levels (Log_10_ TPM).

### Genes under positive selection

Branch site model in the codeml program with the following parameters was used to estimate the dN/dS substitution rate (ω value): positive model: null model: model = 2, NSsites = 2, fix_omega = 1, omega = 1; model = 2, NS sites = 2, fix_omega = 0, omega = 1. A foreground branch was specified as a branch of WT05. Likelihood ratio test (LRT) was used to determine the presence of positive selection in the foreground branch. LRT was calculated according to the following formula: LRT = 2 × |Pos_lnL-Null_lnL|. The significance value (*P* value) was calculated by the chi-square test, which was conducted by chi2 in the PAML package (v4.9) [97], and the degree of freedom was set to 2. In addition, PSGs were defined when the *P* value was less than 0.05, and there has to be at least one site that has a high probability of being positively selected (*P* ≥ 0.95) according to the Bayes empirical Bayes (BEB) test. The functional annotation of PSGs in WT05 was also carried out using the same method as for gene annotation.

### Sequence alignment and variant detection

Reads from each of wild and cultivated castor samples were aligned to the wild castor genome WT05. The same pipeline and parameters as previous publication [17] were used to call variants. The SAMtools program (v1.1) [98] filtered the low-quality (MQ < 20) reads. Picard Tools (http://broadinstitute.github.io/picard/, v1.118) were used to coordinate, sort, and index the sequences. SNP calling was conducted using Genome Analysis Toolkit (GATK, v3.2–2) [99]. Then, the SNP calling results were filtered using the following parameters: QD < 2.0 || MQ < 40.0 || FS > 60.0 || MQRankSum < -12.5 || Read-PosRankSum < -8.0 -clusterSize 3 -clusterWindowSize 10, InDel: QD < 2.0 || FS > 200.0 || ReadPosRankSum < -20.0. Next, GATK with the following parameters “-emitRefConfidence GVCF -variant_index_type LINEAR -variant_index_parameter 128000” was used to second round of SNP calling, which generated GVCF files for each sample. Finally, merged GVCF-format files were used for population variant calling (GATK-3.4–46) with parameters as follows: -stand_call_conf 30.0 -stand_emit_conf 40.0, SNP: QD < 2.0 || MQ < 40.0 || FS > 60.0 || MQRankSum < -12.5 || ReadPosRankSum < -8.0, InDel:QD < 2.0 || FS > 200.0 || ReadPosRankSum < -20.0. Sanger sequencing was applied to validate the accuracy of SNP sites. Total 75 SNPs were randomly selected for PCR, and 99.71% of them were correctly verified by Sanger sequencing.

### GWAS analysis

An efficient mixed-model association (EMMAX) program [100] was used for association analysis. The significance threshold of the relevant SNP was selected as −Log_10_ *P* value > 6.

### Functional annotation of homologous genes

Functional annotation of the candidate genes was based on the functions of their homologs from Euphorbiaceae species and *A. thaliana* via sequence blast.

## Data availability

The assembled genome sequences have been deposited at the NCBI (BioProject: PRJNA589181), and are publicly accessible at https://www.ncbi.nlm.nih.gov/bioproject. Raw data and Assembled data have been separately deposited in the Genome Sequence Archive [101] and the Genome Warehouse [102] at the National Genomics Data Center, Beijing Institute of Genomics, Chinese Academy of Sciences/China National Center for Bioinformation (GSA: CRA003980; GWH: GWHBAUZ00000000), and are publicly accessible at https://ngdc.cncb.ac.cn/. The transcriptome sequencing data have been submitted in the Sequence Read Archive (SRA: SAMN15783672–SAMN15783680), and are publicly accessible at https://www.ncbi.nlm.nih.gov/sra.

## CRediT author statement

**Jianjun Lu:** Formal analysis, Writing - original draft, Supervision, Methodology, Software, Data curation. **Cheng Pan:** Data curation, Investigation. **Wei Fan:** Software, Formal analysis, Visualization, Data curation. **Wanfei Liu:** Software, Formal analysis, Data curation. **Huayan Zhao:** Project administration. **Donghai Li:** Resources, Validation. **Sen Wang:** Software, Formal analysis. **Lianlian Hu:** Conceptualization, Resources. **Bing He:** Software, Formal analysis. **Kun Qian:** Software, Formal analysis. **Rui Qin:** Resources. **Jue Ruan:** Writing - review & editing. **Qiang Lin:** Writing - review & editing, Investigation. **Shiyou Lü:** Writing - review & editing, Validation. **Peng Cui:** Conceptualization, Writing - original draft, Writing - review & editing, Supervision. All authors have read and approved the final manuscript.

## Competing interests

The authors have declared no competing interests.

## Supplementary material

Supplementary Figure S1 **Evaluation of the genome size of WT05 and genome heterozygosity calculation with K-mer 25**

Supplementary Figure S2 **Hi-C reads contact frequency along WT05 chromosomes**

Supplementary Figure S3 **Classification and comparison of genome repeat sequence between WT05 and NSL4733**

Supplementary Figure S4 **Comparison of LTR insertion time of species of Euphorbiaceae**

Supplementary Figure S5 **Collinearity of the genome on 10 chromosomes** Upper yellow lines represent the chromosomes of the WT05 genome, and lower blue lines represent the scaffolds of the NSL4733 genome.

Supplementary Figure S6 **Genome colinear between WT05, NSL4733, and *Arabidopsis thaliana* A.** WT05 and NSL4733. **B.***Arabidopsis thaliana*, WT05, and NSL4733.

Supplementary Figure S7 **Variants between in WT05 and NSL4733 A.** Statistics of SNP and indel variants in the ten chromosomes of WT05 compared with NSL4733. **B.** and **C.** SNP distribution shown by the bar chart (**B**) and pie chart (**C**). D. Classification of structural variations. DUP, inserted duplication; BRK, other inserted sequence; SEQ, rearrangement with another sequence; GAP, gap between two mutually consistent alignments; JMP, rearrangement; INV, rearrangement with inversion.

Supplementary Figure S8 **Correcting truncated genes RcuChr03m1425 and RcuChr01m0783 in cultivated genome NSL4733 based on the transcriptome data from five tissues A.** and **B.** Comparison of gene structures of homologous pairs (RcuChr03m1425 *vs.* 29807.t000005). **C.** and **D.** Comparison of gene structures of homologous pairs (RcuChr01m0783 *vs.* 29736.t000117). The bottom curve represents the alignment sequences. The red dotted boxes indicate the un-annotated exons in the NSL4733 genome. The gray rectangles connecting the green lines represent the transcriptome reads which represent the mapping reads.

Supplementary Figure S9 **Correcting truncated genes RcuChr10m1108 and RcuChr09m1125 in cultivated genome NSL4733 based on the transcriptome data from five tissues A**. and **B.** RcuChr10m1108 is split into 29680.t000078 and 29680.t000079 in the NSL4733 genome. **C.** and **D.** RcuChr09m1125 is split into 30064.t000012 and 30064.t000012 in the NSL4733 genome. In each panel, the upper part shows the depth of transcriptome alignment and the bottom shows the mapping reads. The curves indicate the alignment sequences. The red dotted boxes indicate the un-annotated exons in the NSL4733 genome. The gray rectangles connecting the green line represent the transcriptome reads which represent the mapping reads.

Supplementary Figure S10 **Identification of ricin-related genes A.** Collinear ricin-related genes between WT05 and NSL4733. **B.** Collinearity plot shows the ricin-related genes located on chromosome 8. **C.** Gene trees of 25 ricin-related genes in WT05 genome (left) and 28 ricin-related genes in NSL4733 genome (right). Middle black dotted lines indicate the reciprocal best hit genes. Gray font shows adjacent gene model pairs that may belong to a single pseudogene.

Supplementary Figure S11 **Protein sequence and structure of ricin gene A.** Protein sequences of RIP. The highly diverged peptide in RIP was highlighted in red, and one of the putative active sites located in the highly diverged peptide was shaded in yellow (Tyr129). **B.** 3D protein structure of the ricin A chain (left) and ricin B chain (right). The 3D structure of the highly diverged peptide was highlighted in red.

Supplementary Figure S12 **Ricin-related genes expression pattern and sequence characters A.** Expression profile of the ricin-related genes across different tissues. TPM, transcripts per kilobase million. Data are scaled as Log_10_ TPM. **B.** Motif prediction results of ricin-related genes (motif number: 5).

Supplementary Figure S13 **Part of fatty acid synthesis-associated genes expressed in at least one tissue of castor investigated**

Supplementary Figure S14 **Manhattan and Q-Q plots of nine traits of castor bean** RMFF, ratio of male to female flowers.

Supplementary Figure S15 **An example of gene family expansion A.** Gene tree of the *PSBB* gene family. Red font indicated the expanded *PSBB* genes in the WT05 genome. **B.** Transcriptome alignment of the expanded PSBB genes from different tissues including root, stem, leaf, seed, flower. *PSBB*, photosystem II reaction center protein B; Cas, *M. esculenta* Crantz; Jat, *J. curcas* L.; Rub, *H. brasiliensis*; Ath, *A. thaliana*; Linum, *L. usitatissimum*; Pop, *P. trichocarpa*.

Supplementary Figure S16 **Genomic windows were identified to be associated with selected signals in every chromosome** The horizontal dotted black lines and vertical solid black lines indicate the top 10% selection threshold and the locations of the genes contained in the selection window (10-kb non-overlapping sliding window), respectively.

Supplementary Figure S17 **Population diversity analyses between wild and cultivated castors** Bar plots of the *Fst* (upper) and π_wild_/π_cultivar_ (bottom) values for chromosome 9 between wild and cultivated castor varieties. The horizontal dotted black lines and vertical solid black lines indicate the top 10% selection threshold and the locations of the genes contained in the selection window (10-kb non-overlapping sliding window). **B.** Allelic information of sequence variants in *TPS1* among wild and cultivated castors. Upper part shows the gene structure and expression abundance across five tissues, and gray columns represent transcriptome alignment depth. The dotted red line marked the positions of allele mutations. Bottom shows the allele frequencies of the causal polymorphisms for *TPS1* in different wild and cultivated castor varieties. The numbers in the pie chart represent the number of allele variations at the corresponding location in the wild (total 26) or cultivated (total 159) population. Here, only a portion of the cultivated castor samples is showed (more detailed information is provided in Table S29). -/- indicates allele missing.

Supplementary Figure S18 **The trends between polishing rounds and genome assembly quality A.** Changes in total genome size. **B.** Changes in N50 length.

Supplementary Table S1 **Comparison of genome repeat sequences among species of Euphorbiaceae**

Supplementary Table S2 **Statistics of Nanopore raw data**

Supplementary Table S3 **Statistics of Illumina paired-end and Hi-C raw reads**

Supplementary Table S4 **Assessing WT05 and NSL4733 genome completeness using BUSCO**

Supplementary Table S5 **Mapping ratio of the transcription sets of different tissues of castor**

Supplementary Table S6 **Summary of gene annotation based on different databases**

Supplementary Table S7 **Non-coding RNA genes annotated in the WT05 genome**

Supplementary Table S8 **Statistical information of genomic collinearity at scaffold level**

Supplementary Table S9 **Statistics of genomic alignment between WT05 and NSL4733 genomes (sequence length < 1 kb are excluded)**

Supplementary Table S10 **Statistics of SNPs and indels between WT05 and NSL4733 genomes (sequence length < 1 kb are excluded)**

Supplementary Table S11 **CDS characteristic comparison**

Supplementary Table S12 **Annotation of ricin-related genes in the WT05 genome**

Supplementary Table S13 **Blastp results of the full-length protein sequence of RIP in NR database**

Supplementary Table S14 **Identified potential genes associated with fatty acid synthesis**

Supplementary Table S15 **Primers used for SNP verification**

Supplementary Table S16 **SNPs significantly associated with the nine traits of castor (*P* < 1E−06)**

Supplementary Table S17 **Gene family expansion in WT05 compared to NSL4733 (*P* < 0.01)**

Supplementary Table S18 **GO enrichment of expanded gene families in WT05 compared to NSL4733 genome (*P* < 0.01)**

Supplementary Table S19 **Gene family expansion in WT05 compared to other seven species (*P* < 0.05)**

Supplementary Table S20 **GO enrichment of expanded gene families in WT05 compared to other seven species (*P* < 0.05)**

Supplementary Table S21 **KEGG enrichment of PSGs in the WT05 genome (ω > 1, *P* < 0.05)**

Supplementary Table S22 **GO enrichment of PSGs in the WT05 genome (ω > 1, *P* < 0.05)**

Supplementary Table S23 **Group of PSGs associated with DNA repair and light response**

Supplementary Table S24 **PSGs associated DNA repair identified in other species**

Supplementary Table S25 **Group of PSGs potentially involved in stress responses (Ka/Ks > 1)**

Supplementary Table S26 **Sample IDs of wild and cultivated germplasms**

Supplementary Table S27 **Genomic windows associated with selected signals (top 10% of *Fst* values and top 10% of π_wild_/π_cultivar_ values)**

Supplementary Table S28 **Candidate genes undergone selective pressure (*Fst* and π_wild_/π_cultivar_ at top 10% selection threshold)**

Supplementary Table S29 **SNP variants between wild and cultivated accessions**

Supplementary Table S30 **Comparison of the results of different software assemblies**

Supplementary Table S31 **RNA-seq sample list**

Supplementary Table S32 **Blastp results of the homologous genes in *Arabidopsis thaliana***
