# Supplementary figures and images for: A Chromosome-level Genome Assembly of Wild Castor Provides New Insights into its Adaptive Evolution in Tropical Desert
Source: Genomics Proteomics Bioinformatics. 2021 Jul 30;20(1):42–59. doi: 10.1016/j.gpb.2021.04.003 (PMC9510866; doi:10.1016/j.gpb.2021.04.003)

## GenomeScope Profile

len:318,131,780bp uniq:68.7% het:0.337% kcov:20 err:0.0943% dup:1.34% k:25

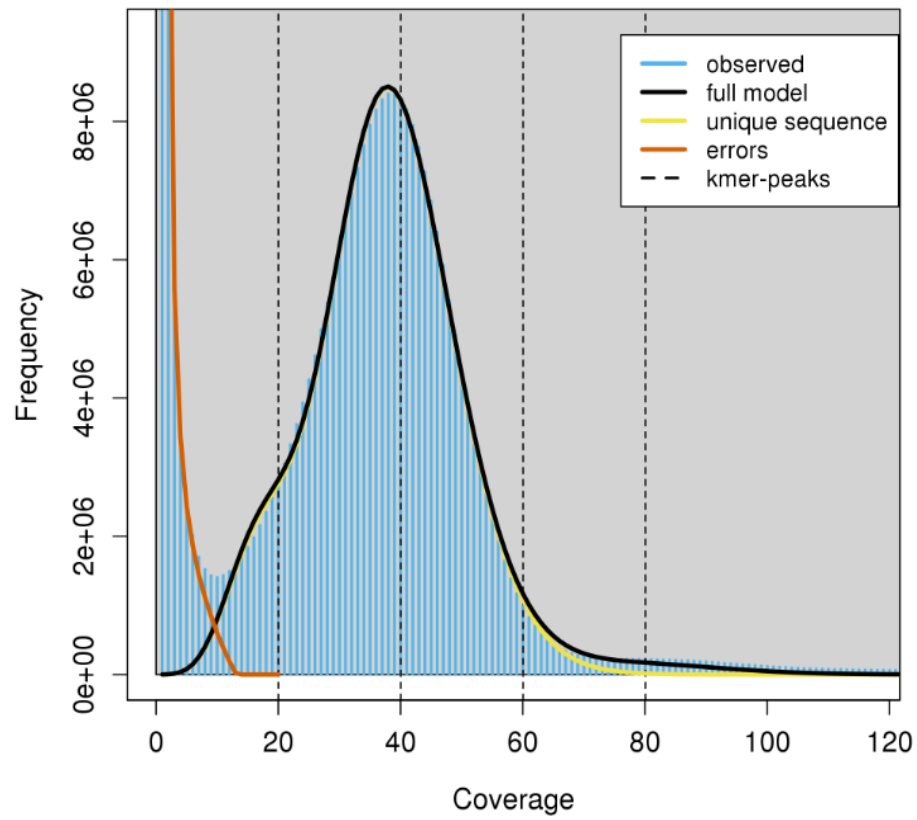

Supplement: Supplementary Figure S1 — Evaluation of the genome size of WT05 and genome heterozygosity calculation with K-mer 25 [file mmc1.pdf]

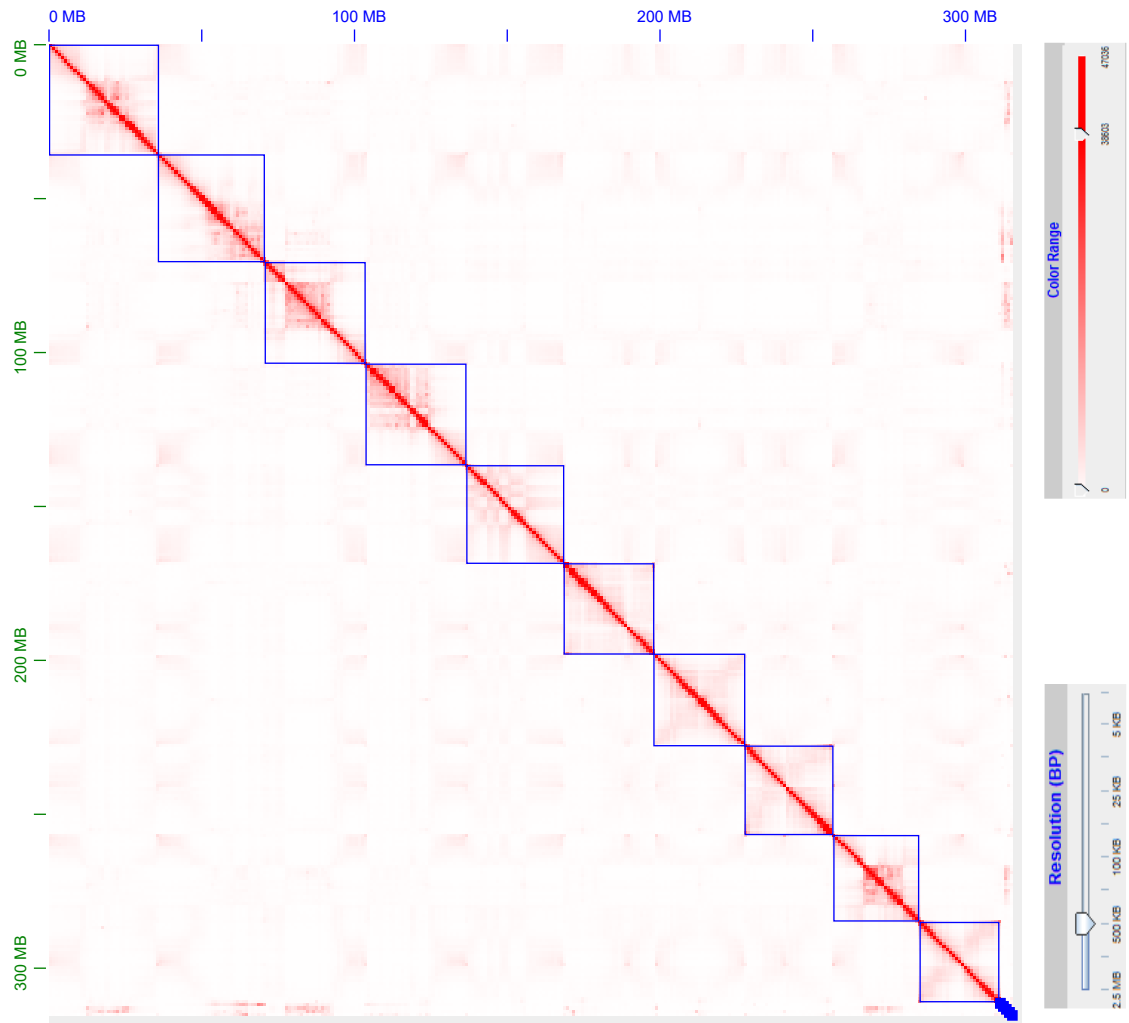

Supplement: Supplementary Figure S2 — Hi-C reads contact frequency along WT05 chromosomes [file mmc2.pdf]

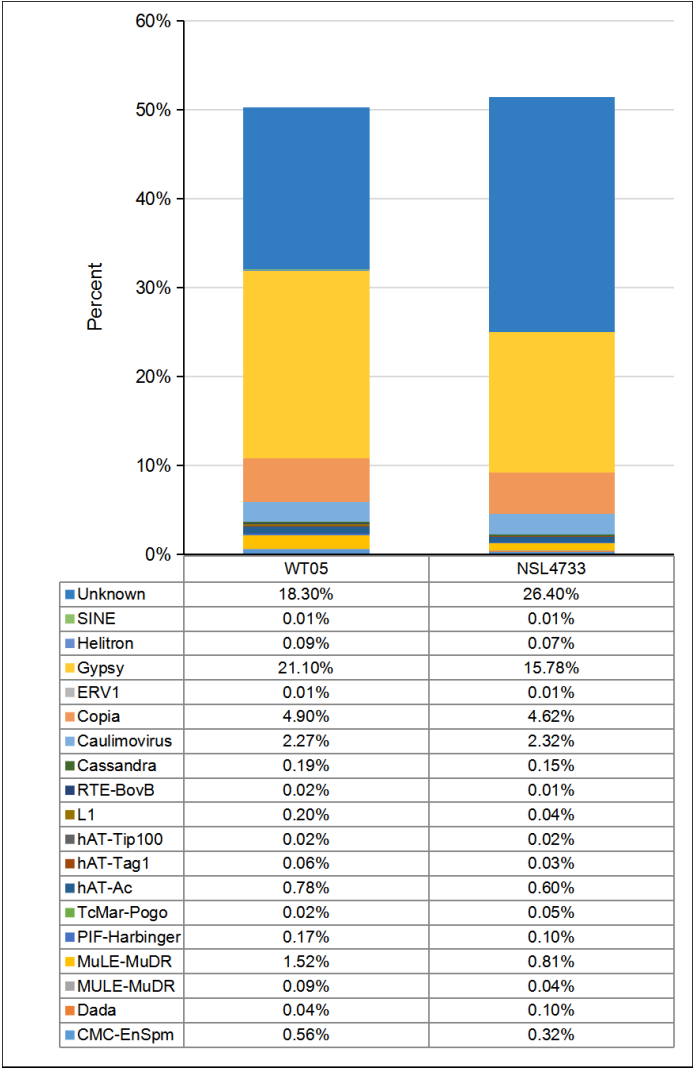

Supplement: Supplementary Figure S3 — Classification and comparison of genome repeat sequence between WT05 and NSL4733 [file mmc3.pdf]

LTR Insertion time estimation

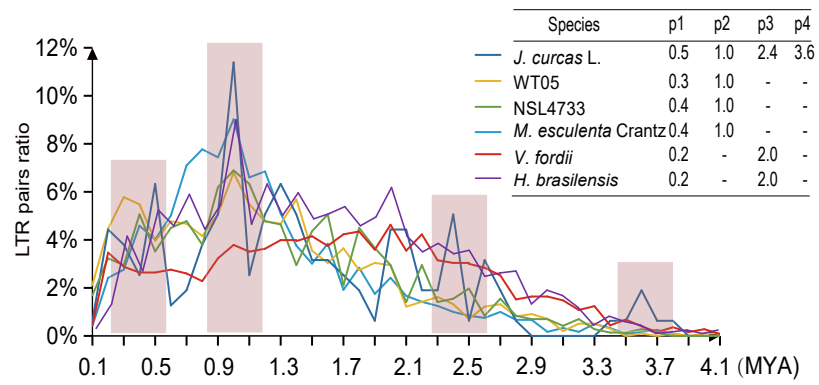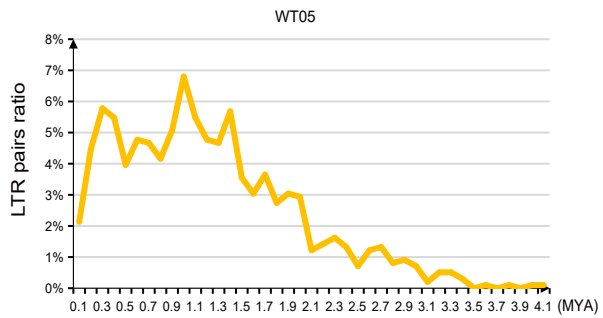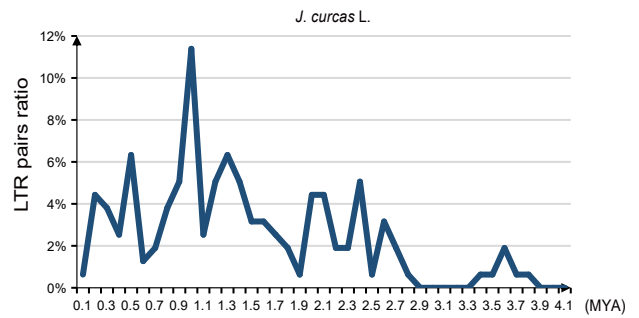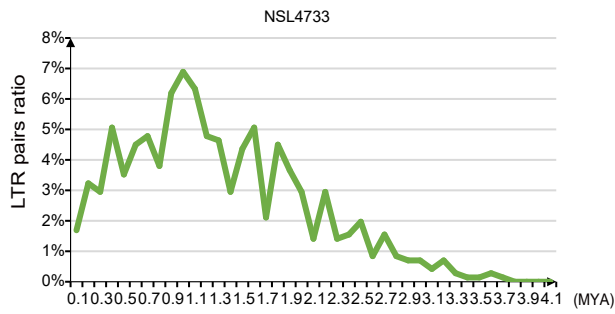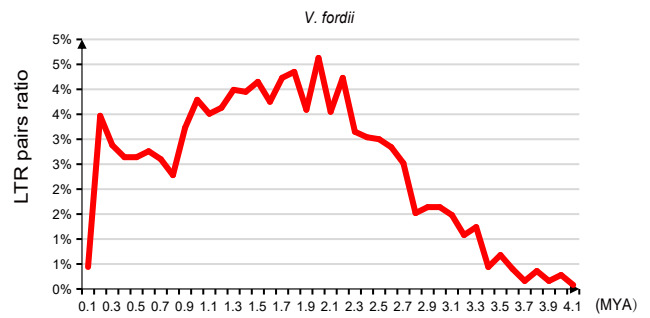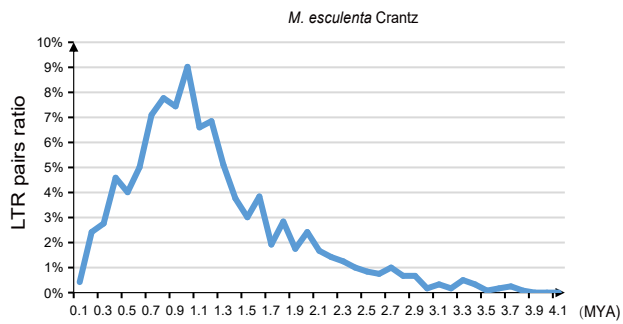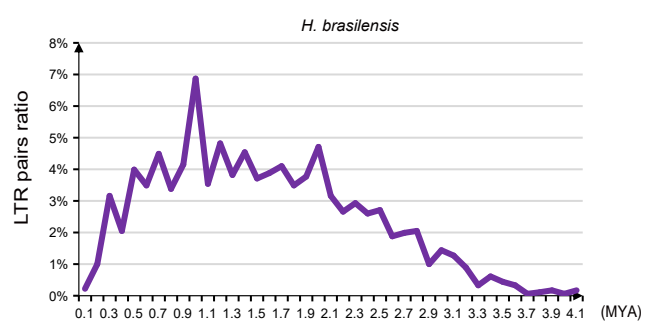

Supplement: Supplementary Figure S4 — Comparison of LTR insertion time of species of Euphorbiaceae [file mmc4.pdf]

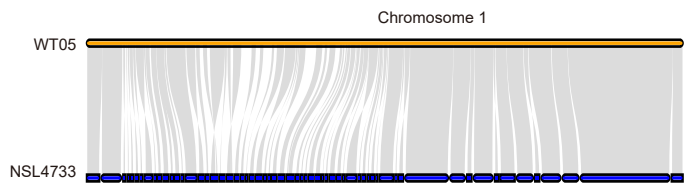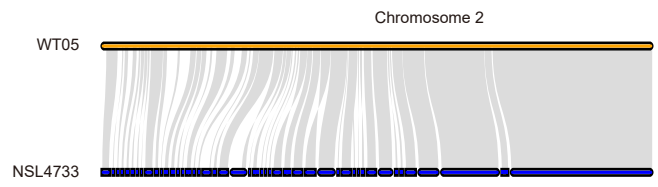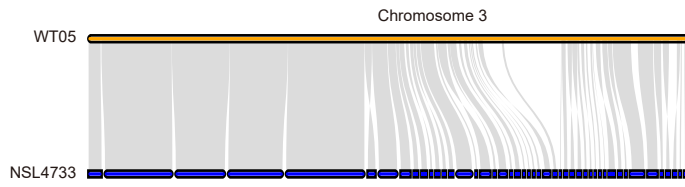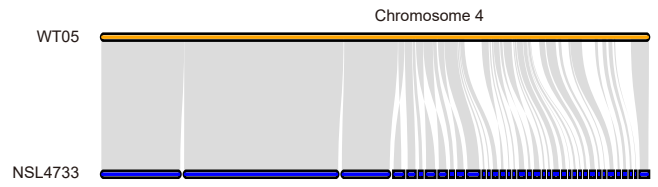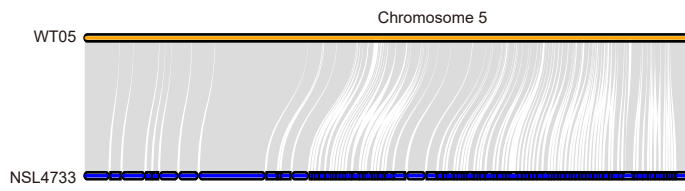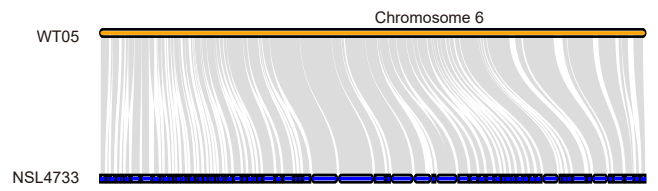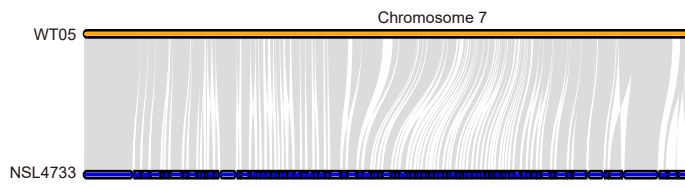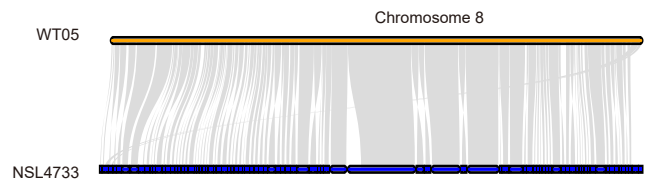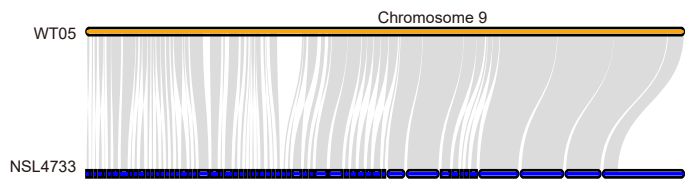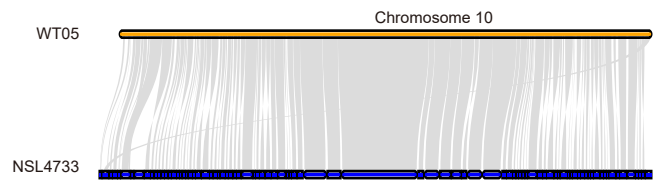

Supplement: Supplementary Figure S5 — Collinearity of the genome on 10 chromosomes Upper yellow lines represent the chromosomes of the WT05 genome, and lower blue lines represent the scaffolds of the NSL4733 genome. [file mmc5.pdf]

**A**

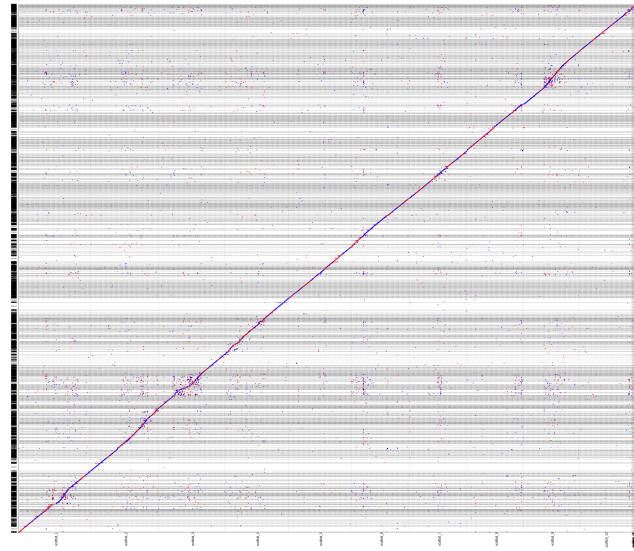

**B**

*A. thaliana*

WT05

NSL4733

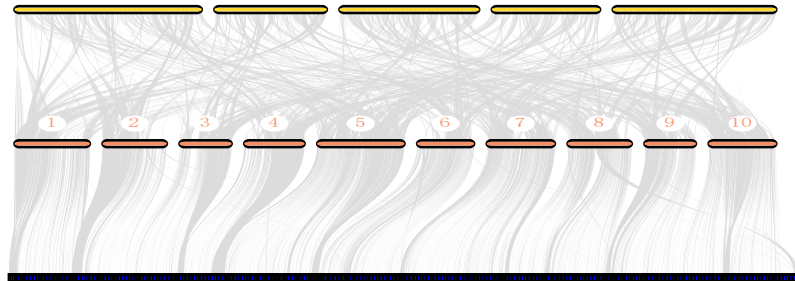

Supplement: Supplementary Figure S6 — Genome colinear between WT05, NSL4733, and Arabidopsis thaliana A. WT05 and NSL4733. B.Arabidopsis thaliana, WT05, and NSL4733. [file mmc6.pdf]

A

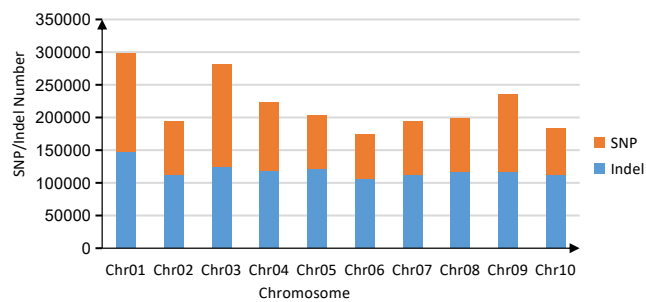

B

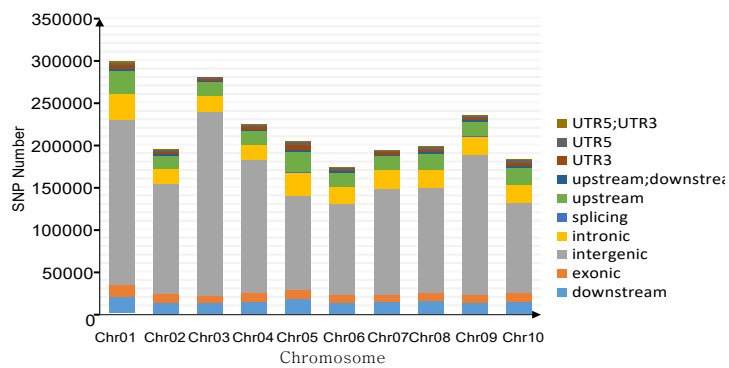

C

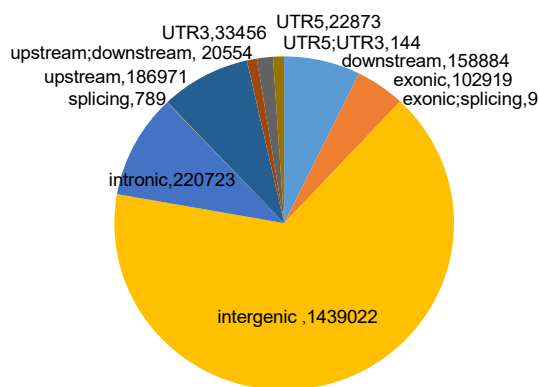

D

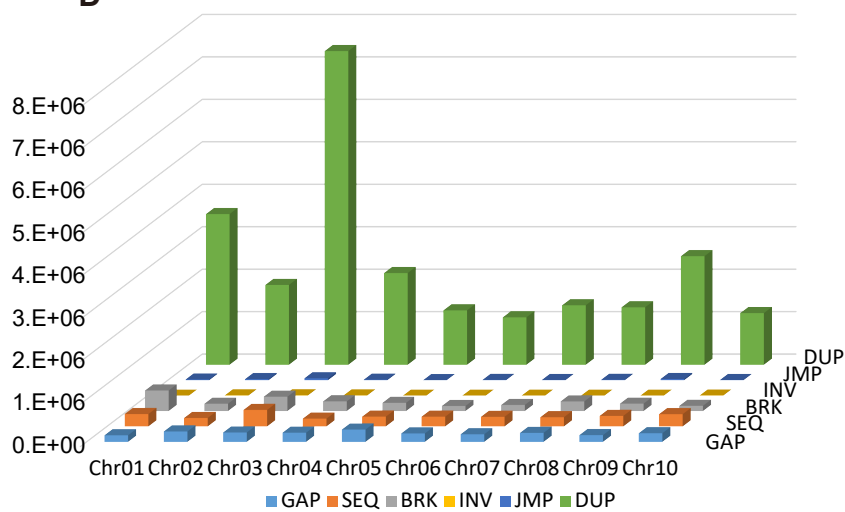

Supplement: Supplementary Figure S7 — Variants between in WT05 and NSL4733 A. Statistics of SNP and indel variants in the ten chromosomes of WT05 compared with NSL4733. B. and C. SNP distribution shown by the bar chart (B) and pie chart (C). D. Classification of structural variations. DUP, inserted duplication; BRK, other inserted sequence; SEQ, rearrangement with another sequence; GAP, gap between two mutually consistent alignments; JMP, rearrangement; INV, rearrangement with inversion. [file mmc7.pdf]

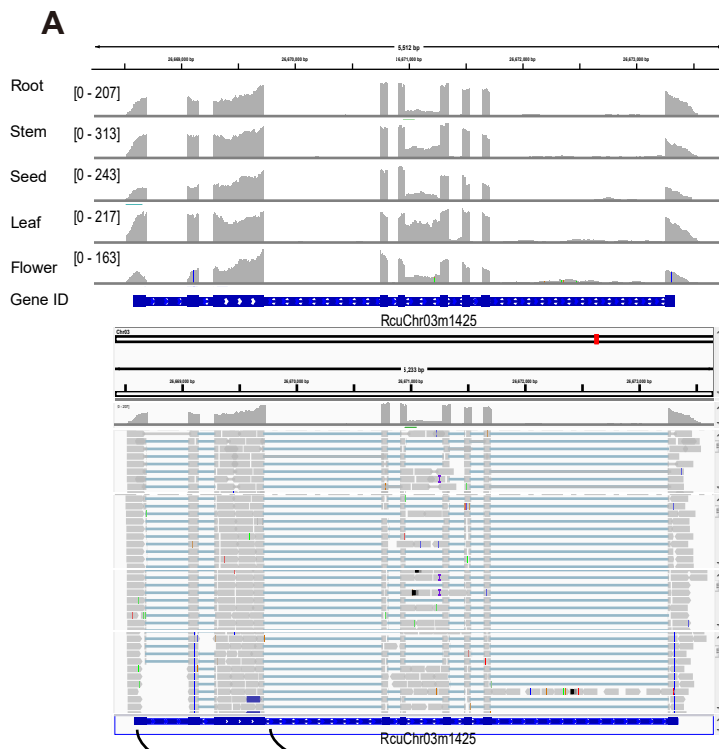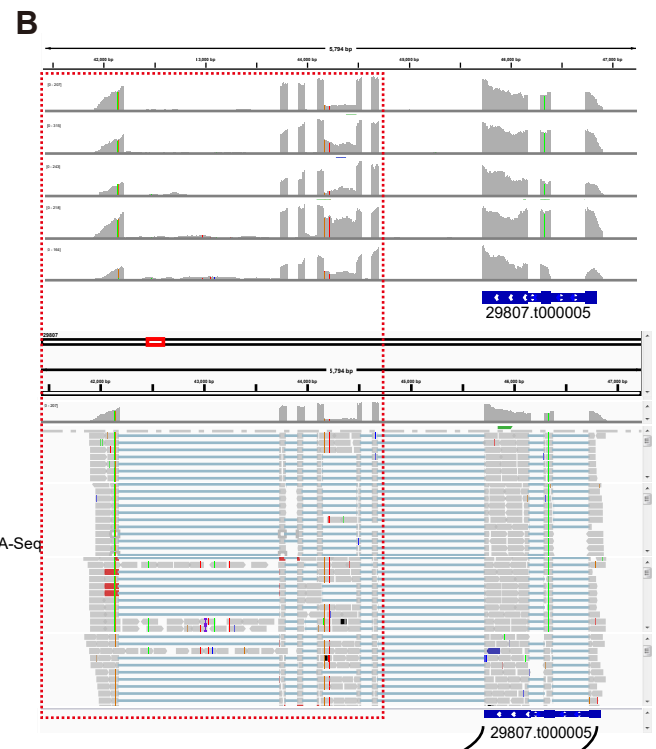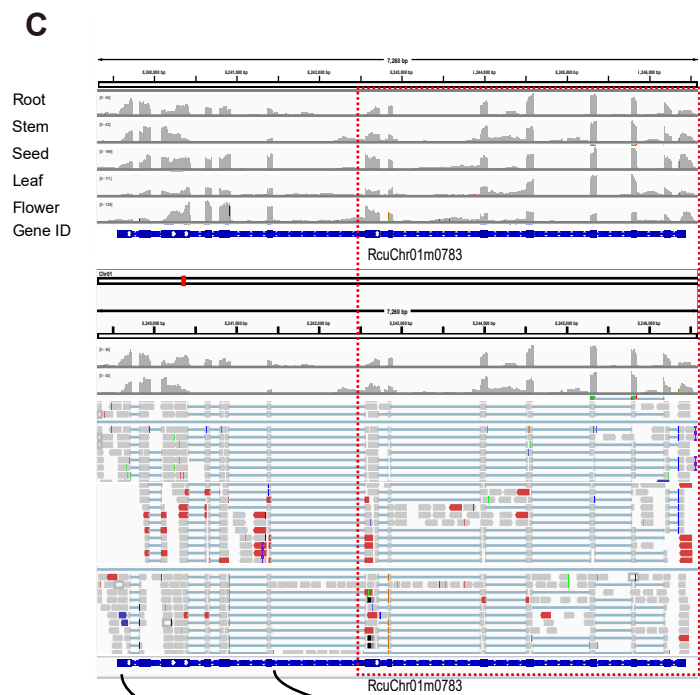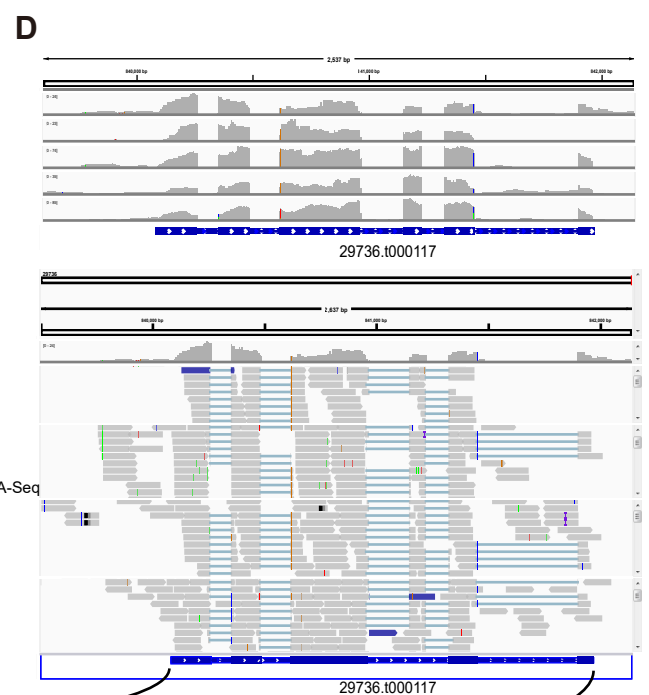

Supplement: Supplementary Figure S8 — Correcting truncated genes RcuChr03m1425 and RcuChr01m0783 in cultivated genome NSL4733 based on the transcriptome data from five tissues A. and B. Comparison of gene structures of homologous pairs (RcuChr03m1425 vs. 29807.t000005). C. and D. Comparison of gene structures of homologous pairs (RcuChr01m0783 vs. 29736.t000117). The bottom curve represents the alignment sequences. The red dotted boxes indicate the un-annotated exons in the NSL4733 genome. The gray rectangles connecting the green lines represent the transcriptome reads which represent the mapping reads. [file mmc8.pdf]

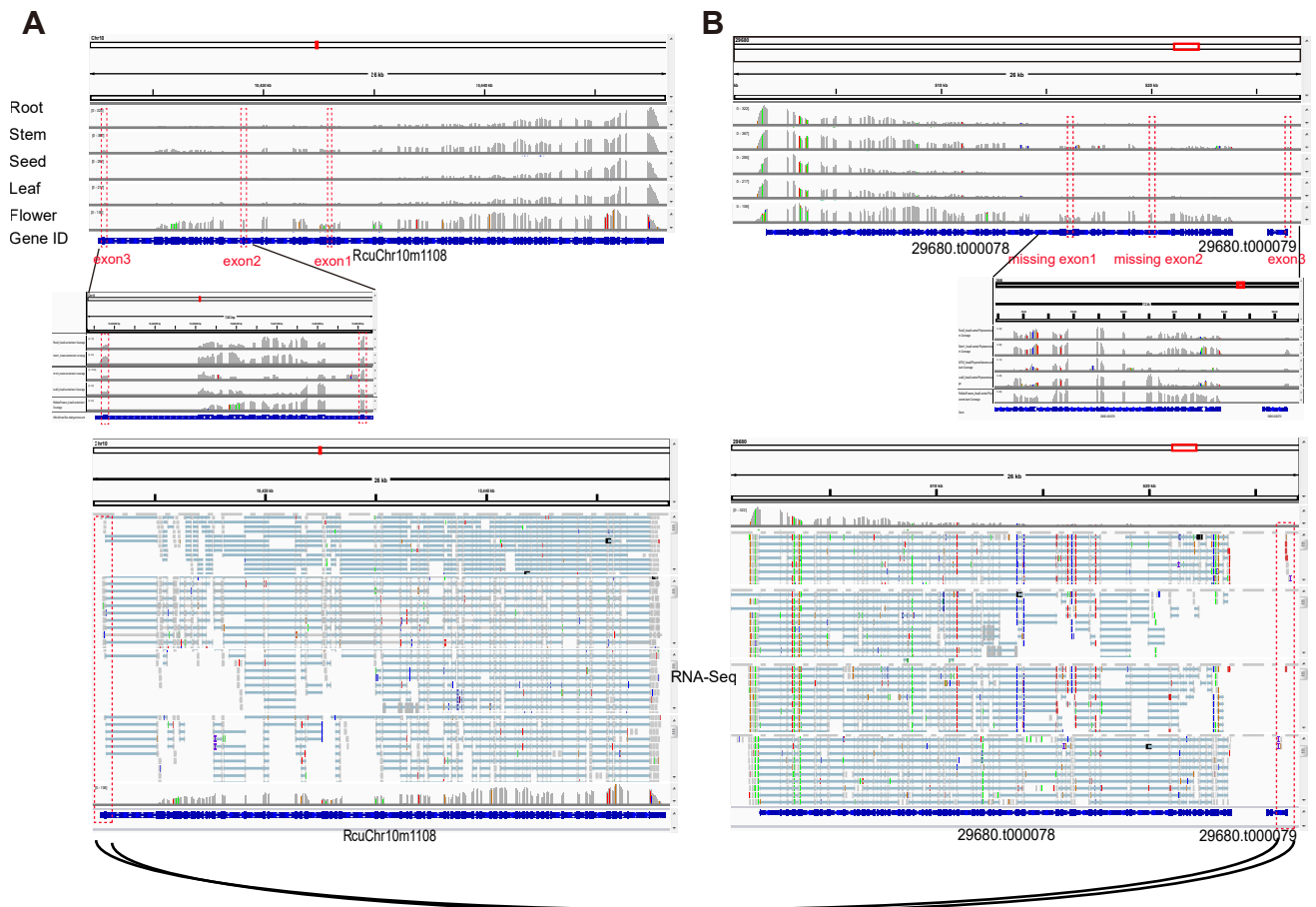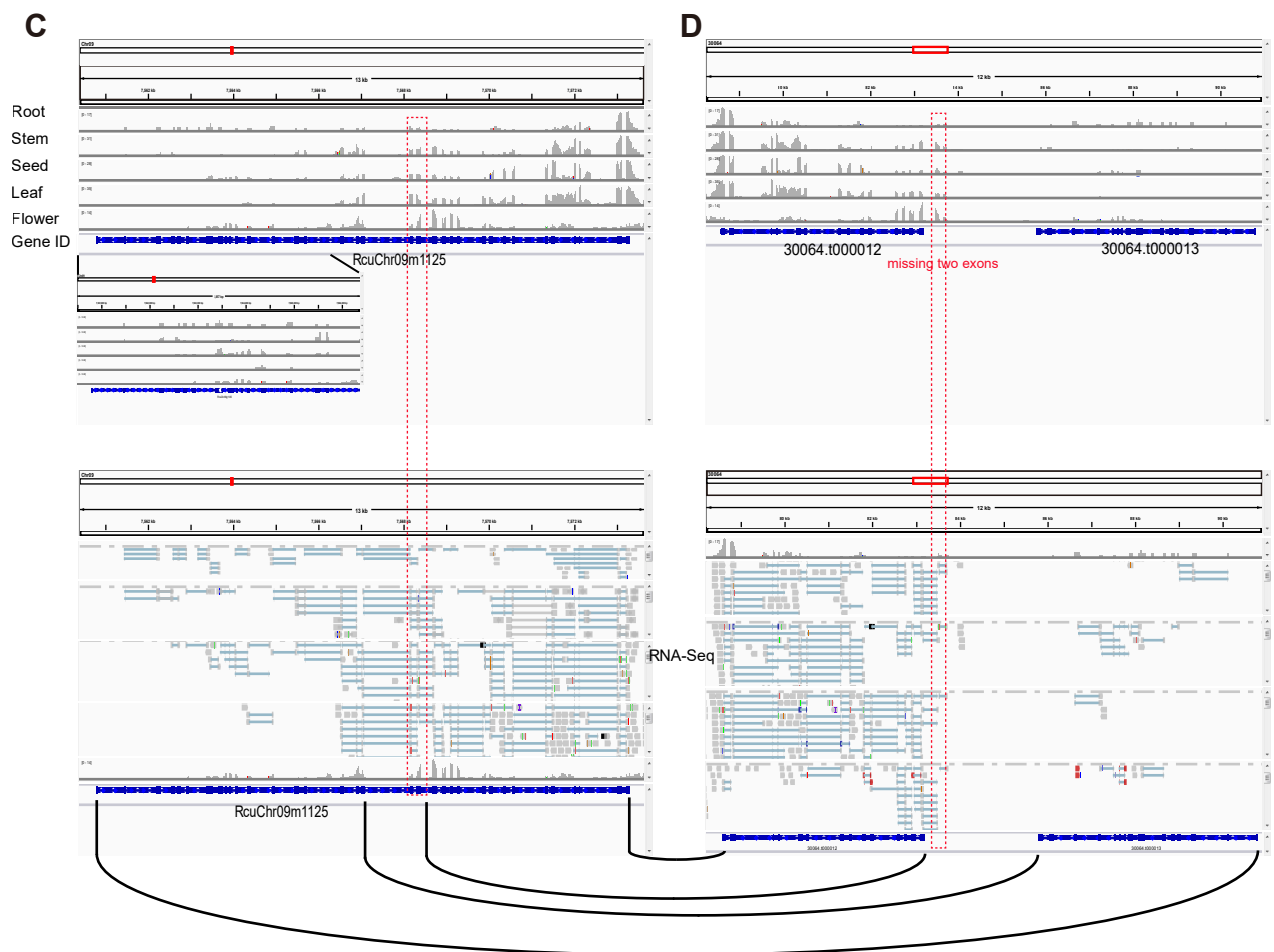

Supplement: Supplementary Figure S9 — Correcting truncated genes RcuChr10m1108 and RcuChr09m1125 in cultivated genome NSL4733 based on the transcriptome data from five tissues A. and B. RcuChr10m1108 is split into 29680.t000078 and 29680.t000079 in the NSL4733 genome. C. and D. RcuChr09m1125 is split into 30064.t000012 and 30064.t000012 in the NSL4733 genome. In each panel, the upper part shows the depth of transcriptome alignment and the bottom shows the mapping reads. The curves indicate the alignment sequences. The red dotted boxes indicate the un-annotated exons in the NSL4733 genome. The gray rectangles connecting the green line represent the transcriptome reads which represent the mapping reads. [file mmc9.pdf]

A

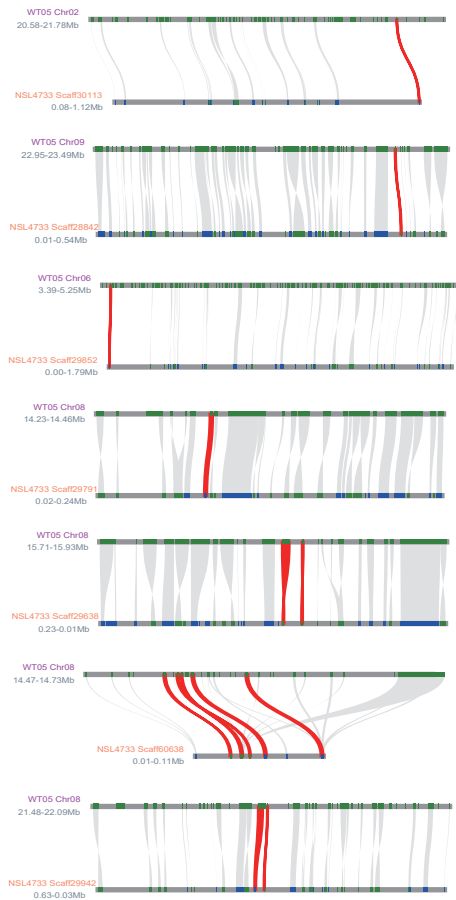

B

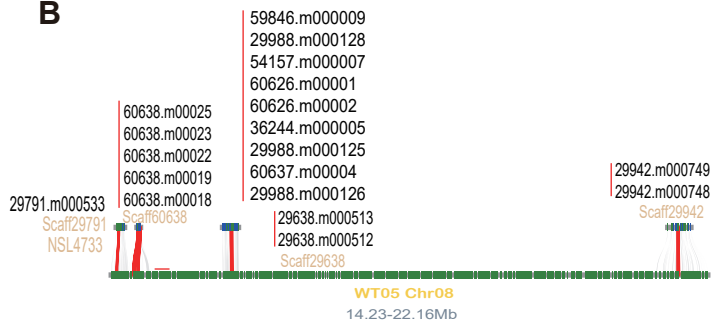

C

Tree scale: 1

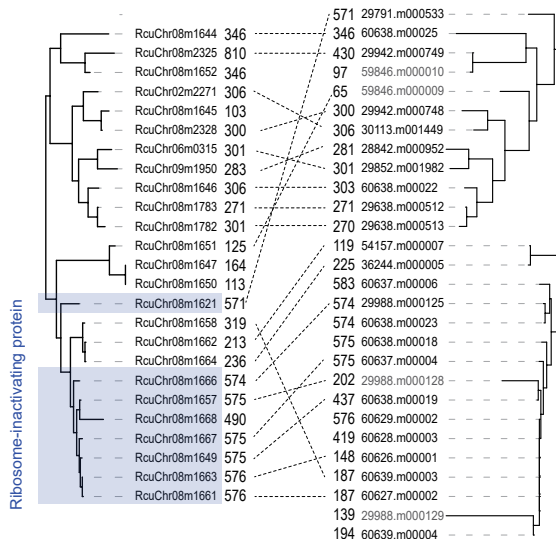

Supplement: Supplementary Figure S10 — Identification of ricin-related genes A. Collinear ricin-related genes between WT05 and NSL4733. B. Collinearity plot shows the ricin-related genes located on chromosome 8. C. Gene trees of 25 ricin-related genes in WT05 genome (left) and 28 ricin-related genes in NSL4733 genome (right). Middle black dotted lines indicate the reciprocal best hit genes. Gray font shows adjacent gene model pairs that may belong to a single pseudogene. [file mmc10.pdf]

**A**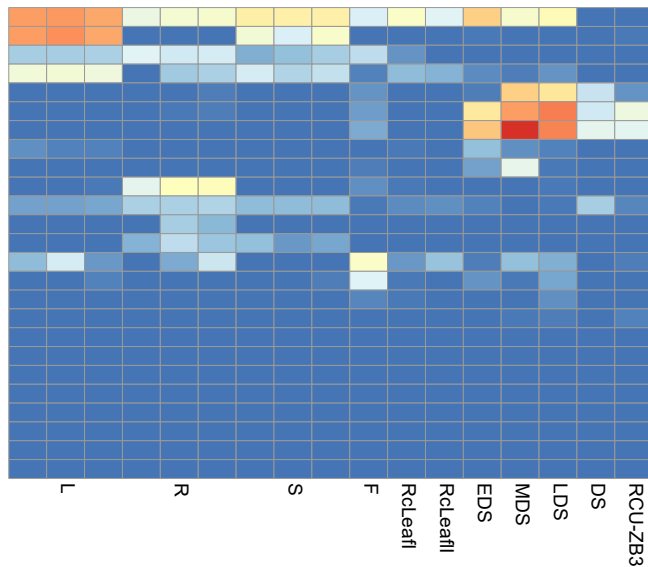**B**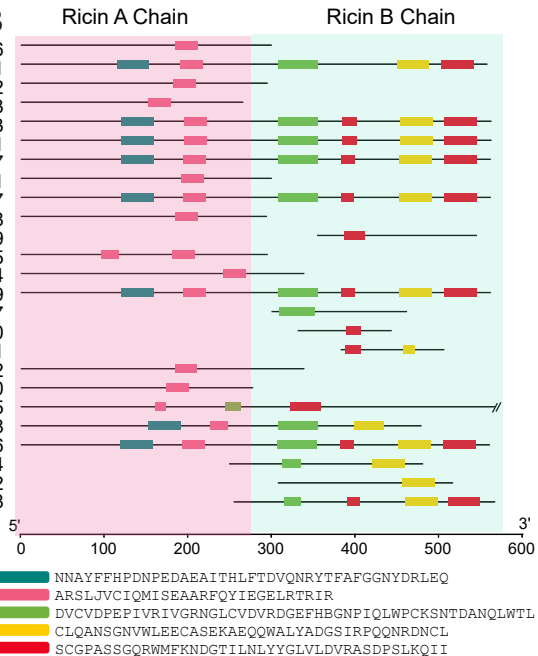

Supplement: Supplementary Figure S12 — Ricin-related genes expression pattern and sequence characters A. Expression profile of the ricin-related genes across different tissues. TPM, transcripts per kilobase million. Data are scaled as Log10 TPM. B. Motif prediction results of ricin-related genes (motif number: 5). [file mmc12.pdf]

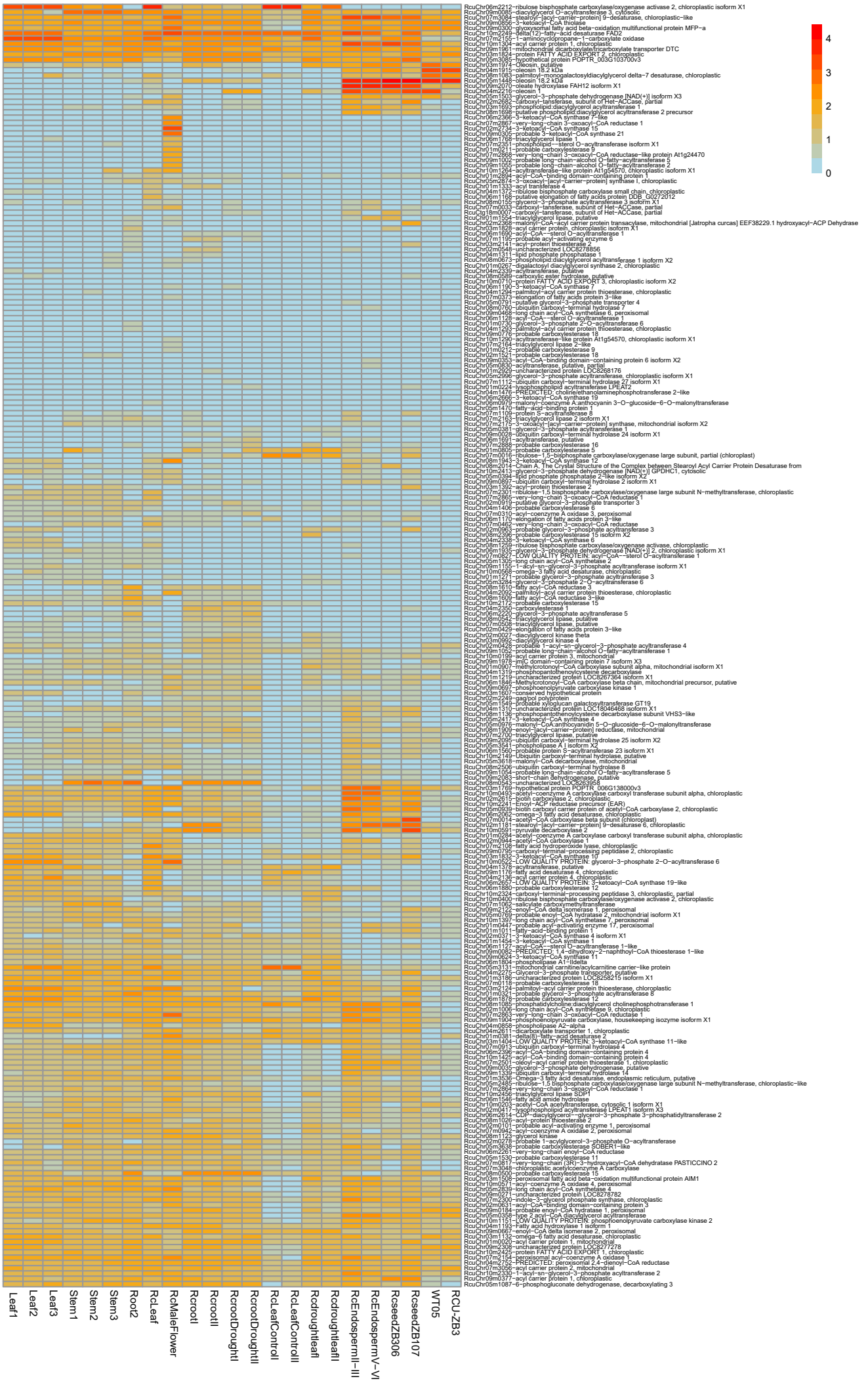

Supplement: Supplementary Figure S13 — Part of fatty acid synthesis-associated genes expressed in at least one tissue of castor investigated [file mmc13.pdf]

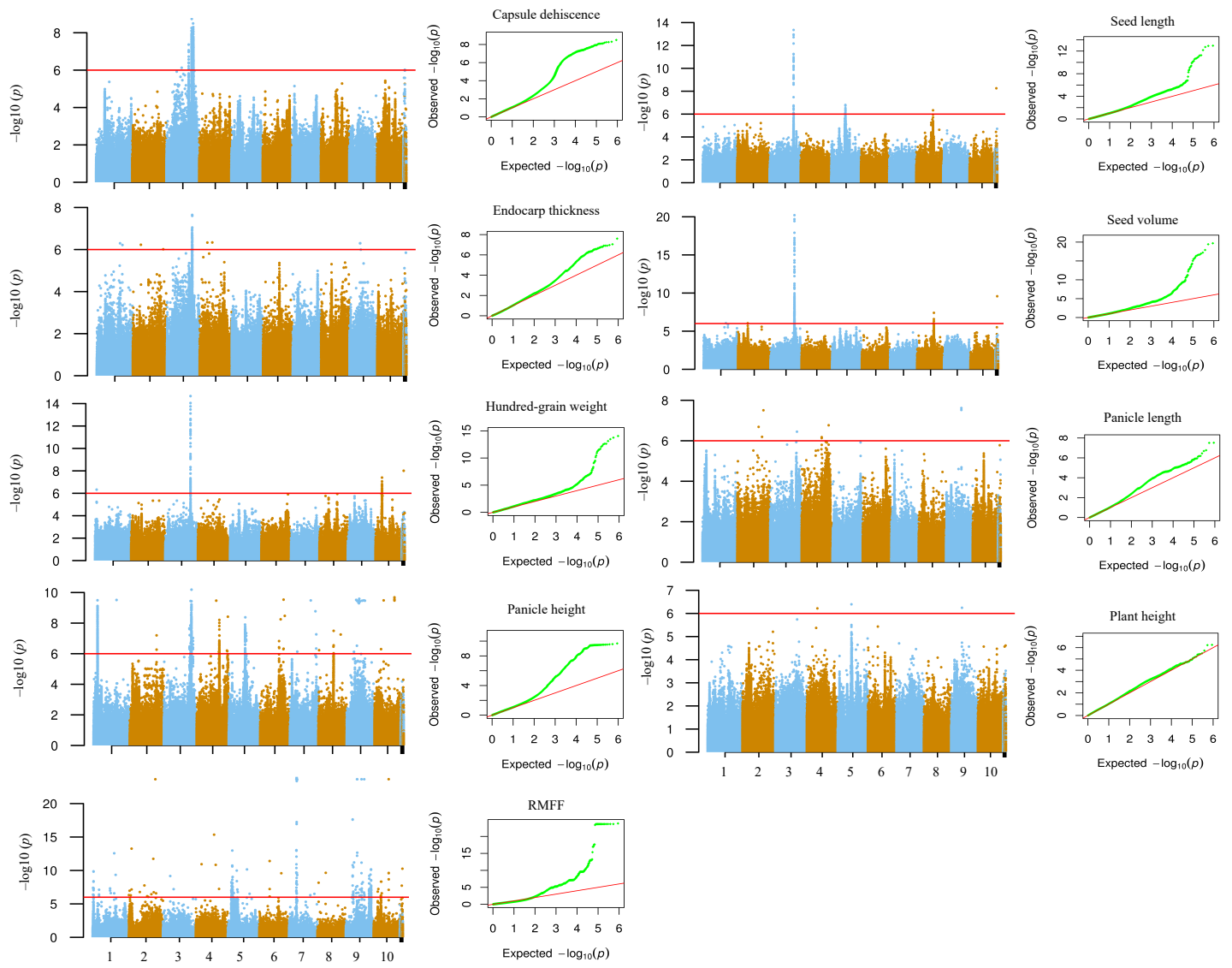

Supplement: Supplementary Figure S14 — Manhattan and Q-Q plots of nine traits of castor bean RMFF, ratio of male to female flowers. [file mmc14.pdf]

**A**

Tree scale: 0.001

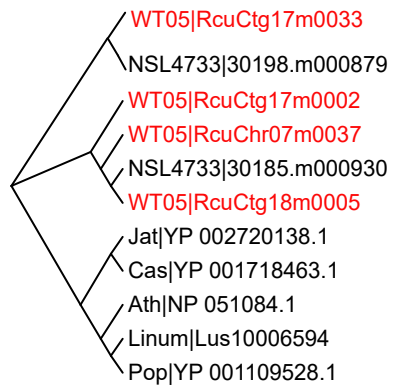

**B**

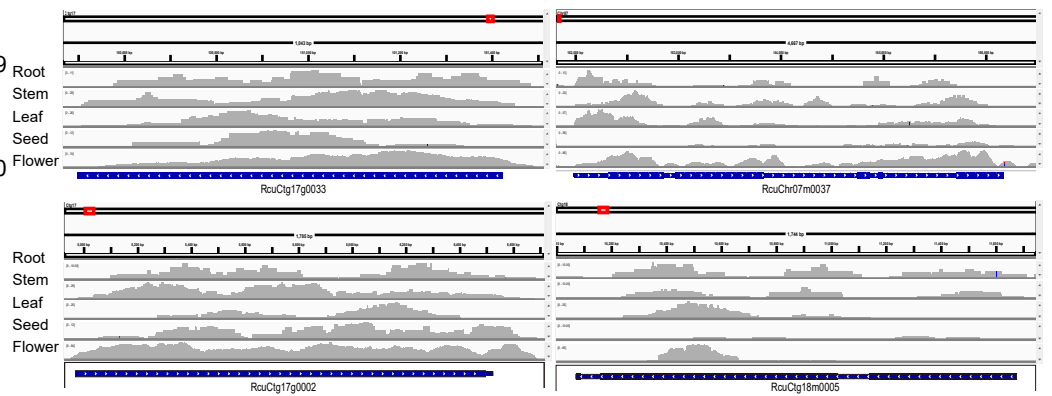

Supplement: Supplementary Figure S15 — An example of gene family expansion A. Gene tree of the PSBB gene family. Red font indicated the expanded PSBB genes in the WT05 genome. B. Transcriptome alignment of the expanded PSBB genes from different tissues including root, stem, leaf, seed, flower. PSBB, photosystem II reaction center protein B; Cas, M. esculenta Crantz; Jat, J. curcas L.; Rub, H. brasiliensis; Ath, A. thaliana; Linum, L. usitatissimum; Pop, P. trichocarpa. [file mmc15.pdf]

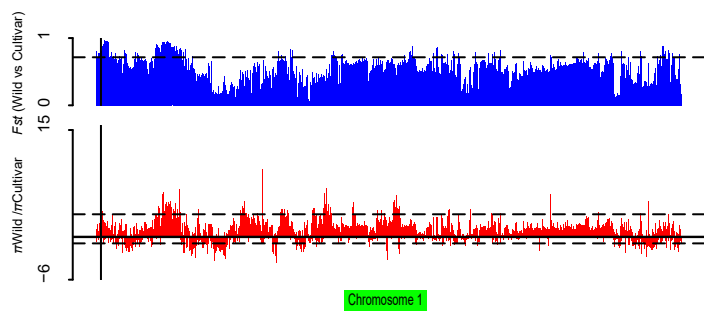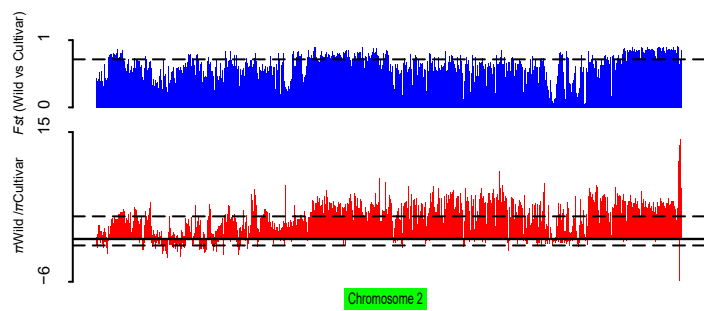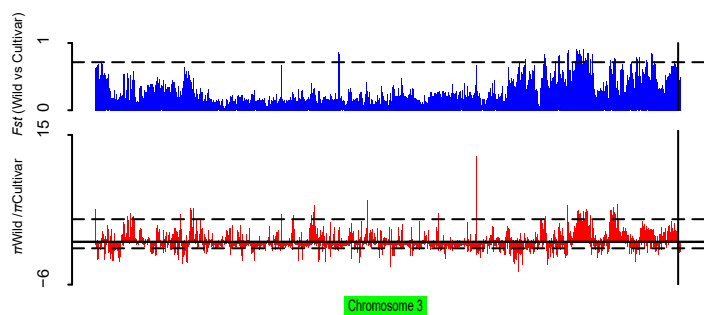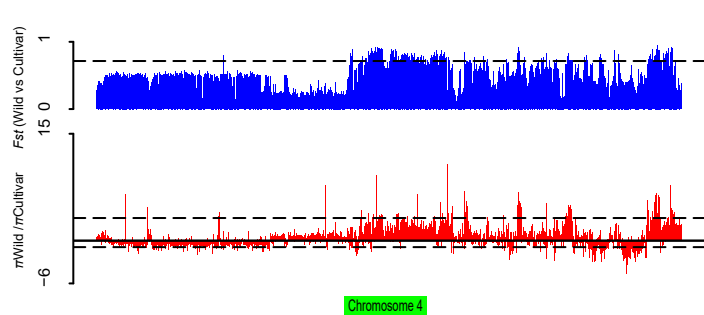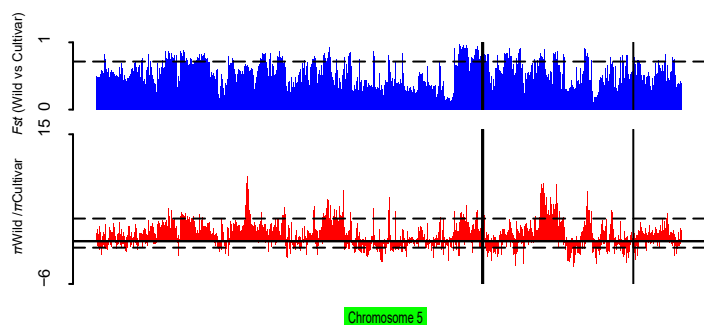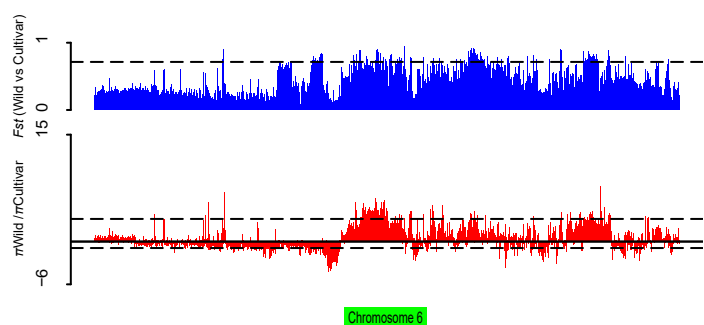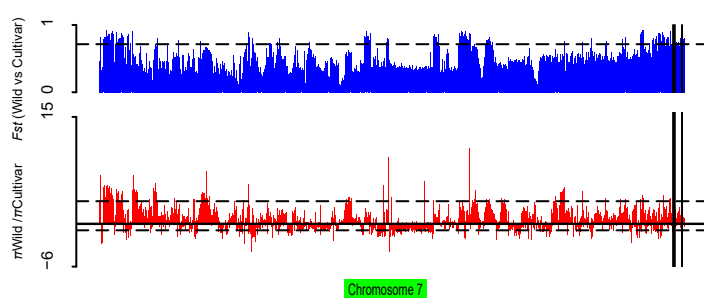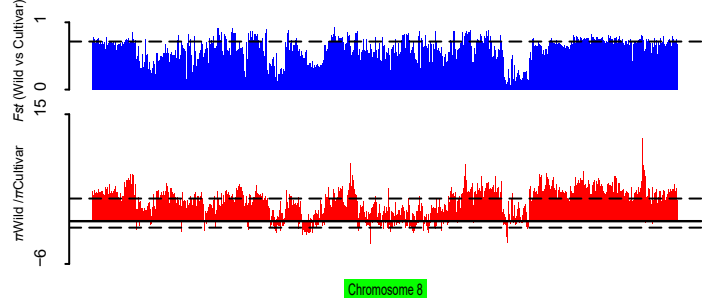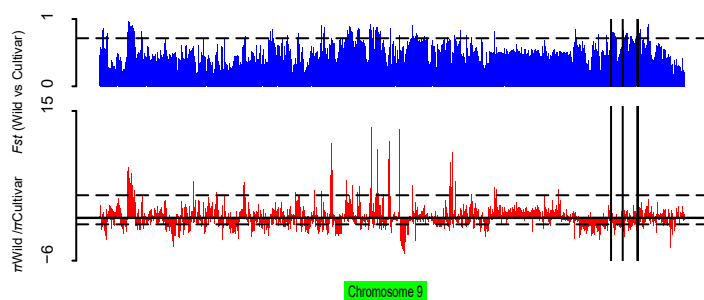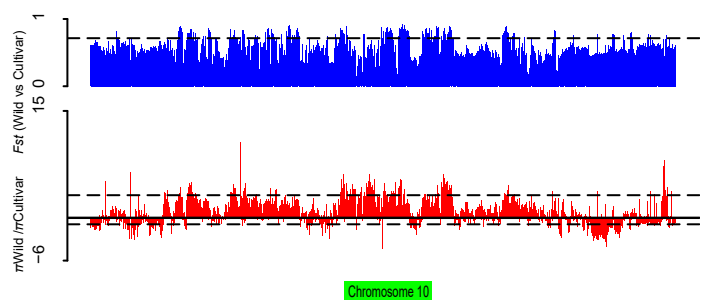

Supplement: Supplementary Figure S16 — Genomic windows were identified to be associated with selected signals in every chromosome The horizontal dotted black lines and vertical solid black lines indicate the top 10% selection threshold and the locations of the genes contained in the selection window (10-kb non-overlapping sliding window), respectively. [file mmc16.pdf]

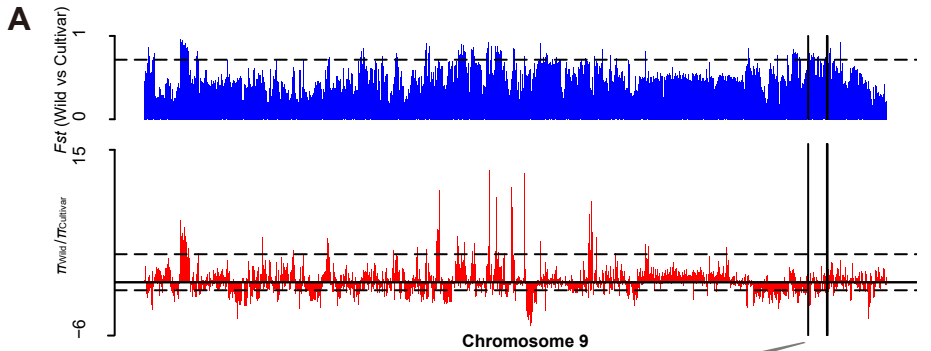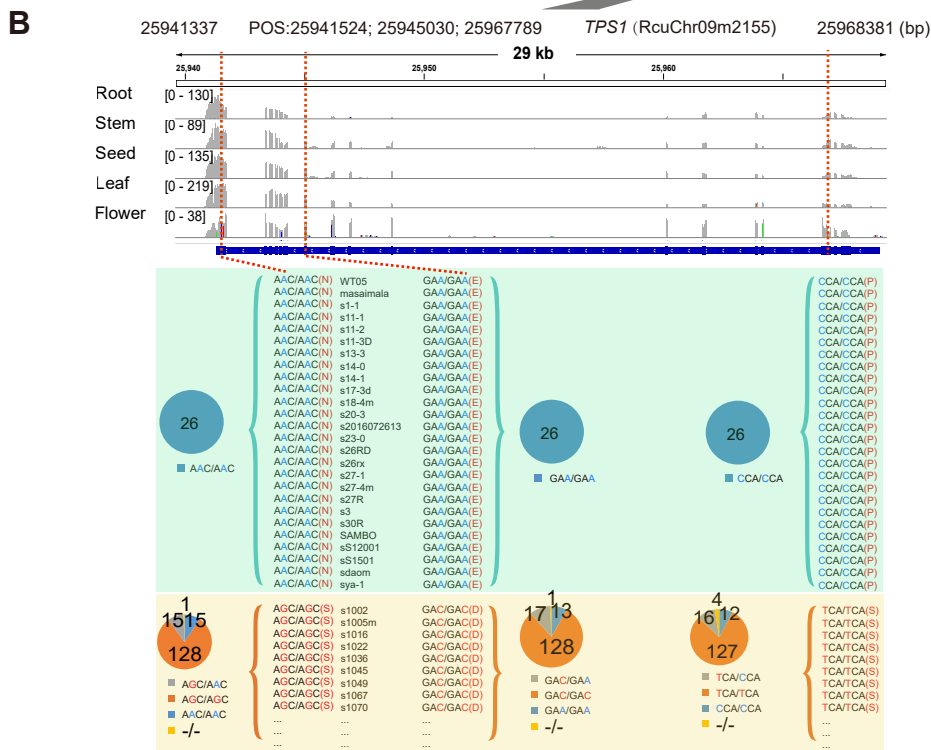

Supplement: Supplementary Figure S17 — Population diversity analyses between wild and cultivated castors Bar plots of the Fst (upper) and πwild/πcultivar (bottom) values for chromosome 9 between wild and cultivated castor varieties. The horizontal dotted black lines and vertical solid black lines indicate the top 10% selection threshold and the locations of the genes contained in the selection window (10-kb non-overlapping sliding window). B. Allelic information of sequence variants in TPS1 among wild and cultivated castors. Upper part shows the gene structure and expression abundance across five tissues, and gray columns represent transcriptome alignment depth. The dotted red line marked the positions of allele mutations. Bottom shows the allele frequencies of the causal polymorphisms for TPS1 in different wild and cultivated castor varieties. The numbers in the pie chart represent the number of allele variations at the corresponding location in the wild (total 26) or cultivated (total 159) population. Here, only a portion of the cultivated castor samples is showed (more detailed information is provided in Table S29). -/- indicates allele missing. [file mmc17.pdf]

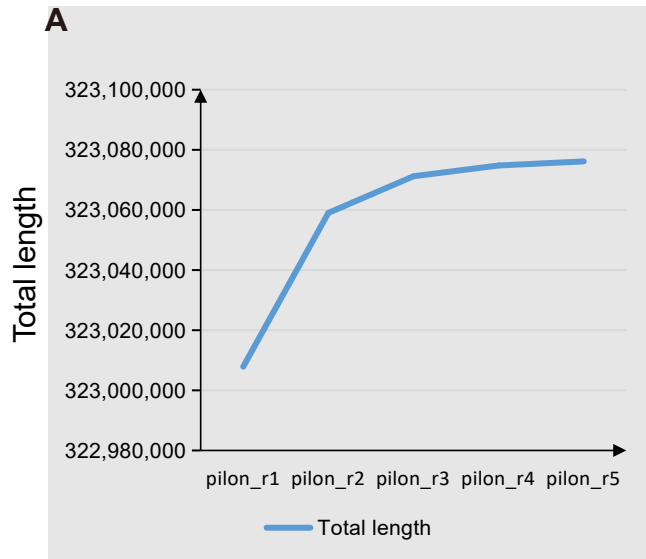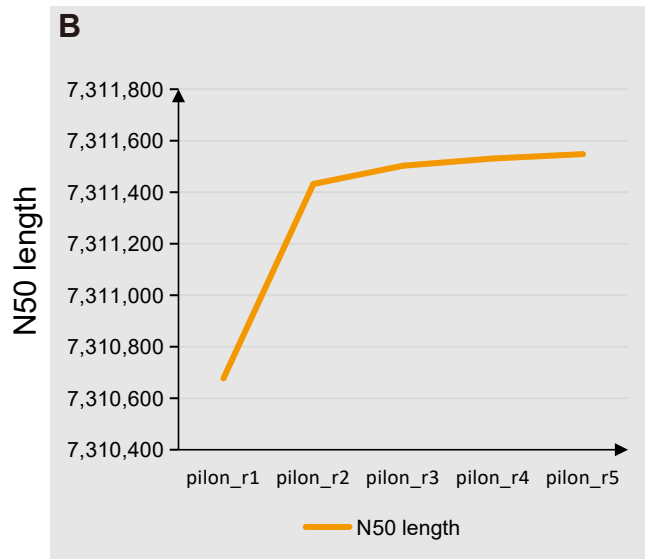

Supplement: Supplementary Figure S18 — The trends between polishing rounds and genome assembly quality A. Changes in total genome size. B. Changes in N50 length. [file mmc18.pdf]
