# Supplementary material for: A Chromosome-level Genome Assembly of Wild Castor Provides New Insights into its Adaptive Evolution in Tropical Desert
Source: Genomics Proteomics Bioinformatics. 2021 Jul 30;20(1):42–59. doi: 10.1016/j.gpb.2021.04.003 (PMC9510866; doi:10.1016/j.gpb.2021.04.003)

A

Ricin A chain:1-277 （101-143）

IFPKQYPIINFTTIFPKQYPIINFTTADATVESYTNFIRAVRSHLTTGGDV
RHEIPVLPNRVGLPISQRFILVELSNHAELSVTLALDVTNAYVVGCRAGNS
AYFFHPDNQEDAEAITHLFTDVQNSFTFAFGGN**Y**DRLEQLGGLRENIELGT
GPLEDAISALYYYSTCGTQIPTLARSFMVCIQMISEAARFQYIEGEMRTRI
RYNRRSAPDPSVITLENSWGRLSTAIQESNQGAFASPIQLQRRNGSKFNVY
DVSILIPIIALMVYRCAPPPQF

12 amino acid link Achain and Bchain:--SLLIRPVVPNFN--

Ricin B chain: 290-552

ADVCMDPEPIVRIVGRNDLCVDVRDGRFHNGNAIQLWPCKSNTDANQLWTL
KRDNTIRSNGKCLTTYGYSPGVYVMIYDCDTAVTDATRWQIWDNGTIINPR
SSLVLAATSGNSGTTLTVQTNIYSVSQGWLPTNNTQPFVTTIVGLYGMCLQ
ANSGQVWLEDCTSEKAEQQWALYADGSIRPQQNRDNCLTTDANIQGTVVKI
LSCGPASSGQRWMFKNDGSILNLYNGLVLDVTRSDPSLKQIIVHPFHGNLN
QIWLPLF

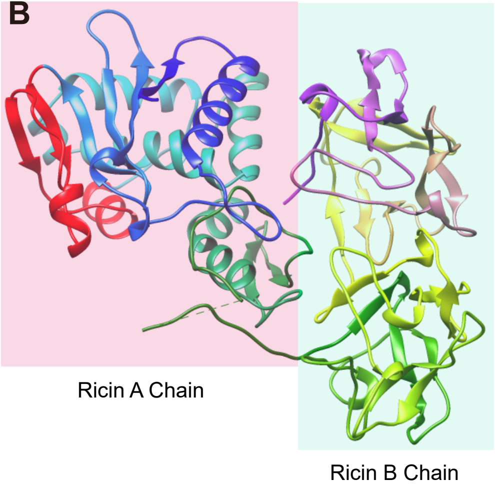

Supplement: Supplementary Figure S11 — Protein sequence and structure of ricin gene A. Protein sequences of RIP. The highly diverged peptide in RIP was highlighted in red, and one of the putative active sites located in the highly diverged peptide was shaded in yellow (Tyr129). B. 3D protein structure of the ricin A chain (left) and ricin B chain (right). The 3D structure of the highly diverged peptide was highlighted in red. [file mmc11.pdf]
